# Supplementary material for: JMY powers dendritogenesis and is regulated by CaM revealing a general, critical principle in neuromorphogenesis
Source: Commun Biol. 2025 May 22;8:784. doi: 10.1038/s42003-025-08208-3 (PMC12098658; doi:10.1038/s42003-025-08208-3)
Supplement: Supplementary file 2 — Supplementary Information [file 42003_2025_8208_MOESM2_ESM.pdf]

# Supplementary Information

## JMY powers dendritogenesis and is regulated by CaM revealing a general, critical principle in neuromorphogenesis

Maja Kühne[1], Anna-Lena Zepernick[1], Britta Qualmann[1#*], Michael Manfred Kessels[1#*], Maryam Izadi-Seitz[1#*]

[1] Institute of Biochemistry I, Jena University Hospital - Friedrich Schiller University Jena, Nonnenplan 2-4, 07743 Jena, Germany

[#] these authors contributed equally

* correspondence should be addressed to B.Q., M.M.K & M.I.-S.

Britta.Qualmann@med.uni-jena.de (OCID 0000-0002-5743-5764), &

Michael.Kessels@med.uni-jena.de (OCID 0000-0001-5967-0744) &

Maryam.Izadi@med.uni-jena.de (OCID 0000-0002-8260-2560)

**Abbreviated title:** JMY powers dendritogenesis and is regulated by CaM

## Supplementary Figure S1

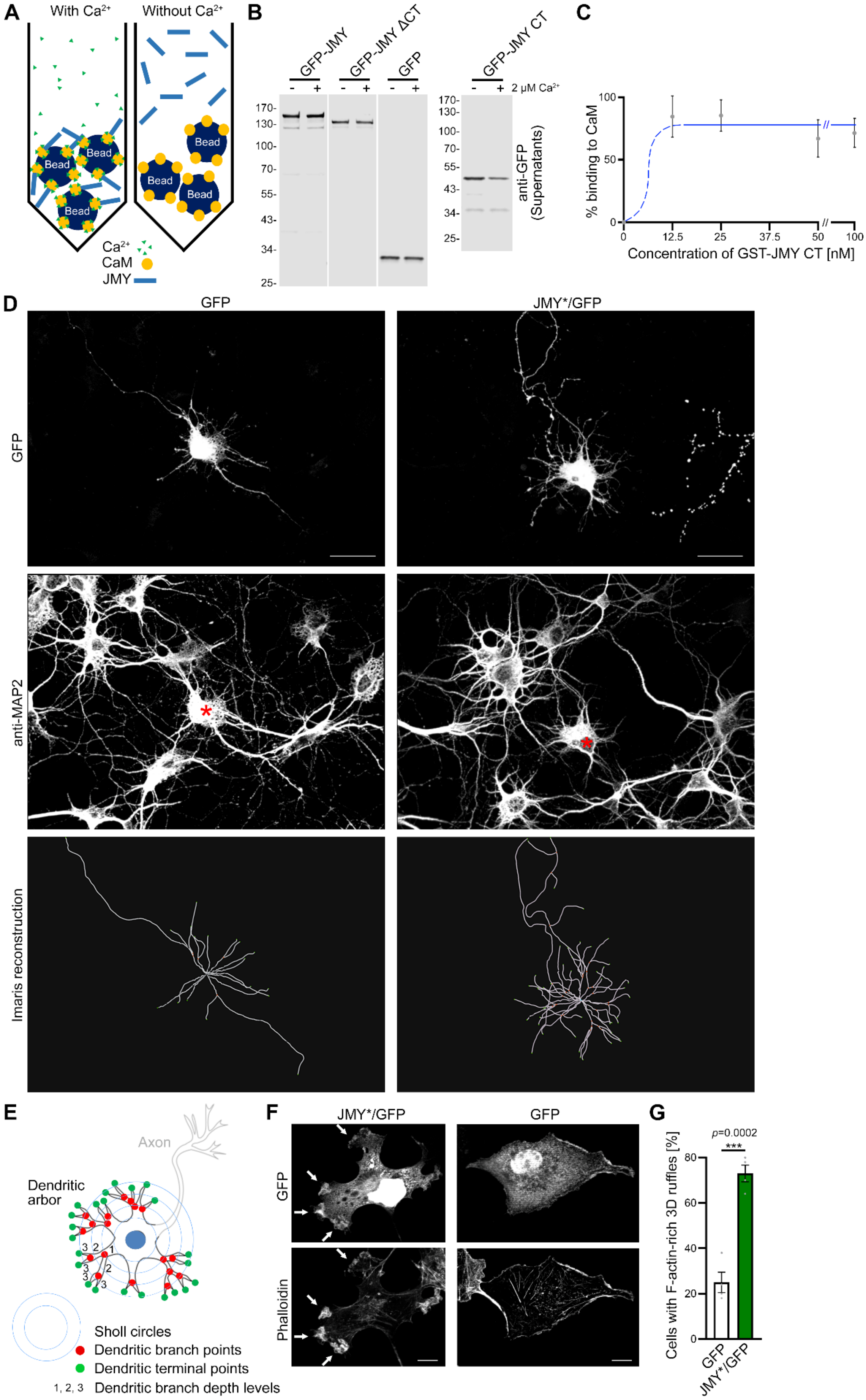

**Supplementary Figure 1. JMY is a positive regulator of F-actin-rich 3D ruffles.**

(**A**) Schematic representation of coprecipitation experiments with CaM immobilized on beads and JMY under either $Ca^{2+}$-containing conditions leading to calcium loading and activation of CaM, which then associates with JMY, or under conditions lacking $Ca^{2+}$ and CaM activation, which do not allow for CaM association and therefore result in JMY remaining in the supernatant during bead precipitation by centrifugation. (**B**) Immunoblot analyses of supernatants from the coprecipitation experiments of GFP-JMY and fragments thereof expressed in L929 cells using a CaM matrix shown in Fig. 1A. The anti-GFP signal of the supernatants confirmed the expression and the stability of GFP and the GFP-fusion proteins in the assay. White lines indicate lanes omitted from the blot (due to change of order). (**C**) Quantitative binding studies with constant amounts of CaM in 20 µM $Ca^{2+}$ and different amounts of GST-JMY CT to determine the $K_D$ of the protein complex formation. Note that even with only 12.5 nM GST-JMY CT (detection limit), the association was still at saturation level, i.e. the binding curve could only be extrapolated (dashed line). Note that due to space constraints, the x-axis had to be broken in order to fit the additional value for 100 nM GST-JMY CT (binding also in saturation, as for 12.5, 25 and 50 nM). (**D**) Additional images corresponding to Fig. 1G. Anti-MAP staining and GPF signals of primary hippocampal neurons transfected with an IRES-containing plasmid driving either just GFP as reporter or the expression of untagged JMY (in an RNAi#1-insensitive form; JMY*) and GFP (JMY*/GFP) at DIV4 and fixed 30 h thereafter. Asterisks mark transfected neurons. Lower panels show 2D representations of 3D morphometric reconstructions by using Imaris software. Note that viewing the images at high magnification also allows for seeing the Imaris-based markings of branch points (red) and terminal points (green). Bars, 30 µm. (**E**) Schematic representation of a developing neuron. Dendritic branch points (red), dendritic terminal points (green), numbers highlighting dendritic branch depth levels and Sholl cirles are marked for illustration of Imaris software-based evaluation parameters (*total dendritic length*, lines of measurement originating

from cell center is not depicted; see Imaris reconstructions in **D** for illustration). (**F,G**) JMY expression promotes the formation of F-actin-rich 3D ruffles. (**F**) MIPs of phalloidin-stained COS-7 cells expressing GFP and untagged JMY* together with GFP as reporter, respectively. Bars, 10 µm. (**G**) Quantitative evaluations of JMY-induced F-actin-rich 3D ruffle formation (arrows) by scoring the percentage of cells with such structures. Data, mean±SEM shown as line plot (**C**) and bar/dot plot (**G**), respectively. (**C**) 0 nM, n=6; 12.5 nM, n=3; 25 nM, n=3; 50 nM, n=6; 100 nM, n=6 binding assays. (**G**) n=4 independent assays. Numerical data and images of uncropped and unedited blots are provided in Supplementary Data 1 and Supplementary Data 2, respectively. Statistical significances, Welch′s t-test (**G**); ***$p<0.001$. For exact $p$ value see figure panels.

## Supplementary Figure S2

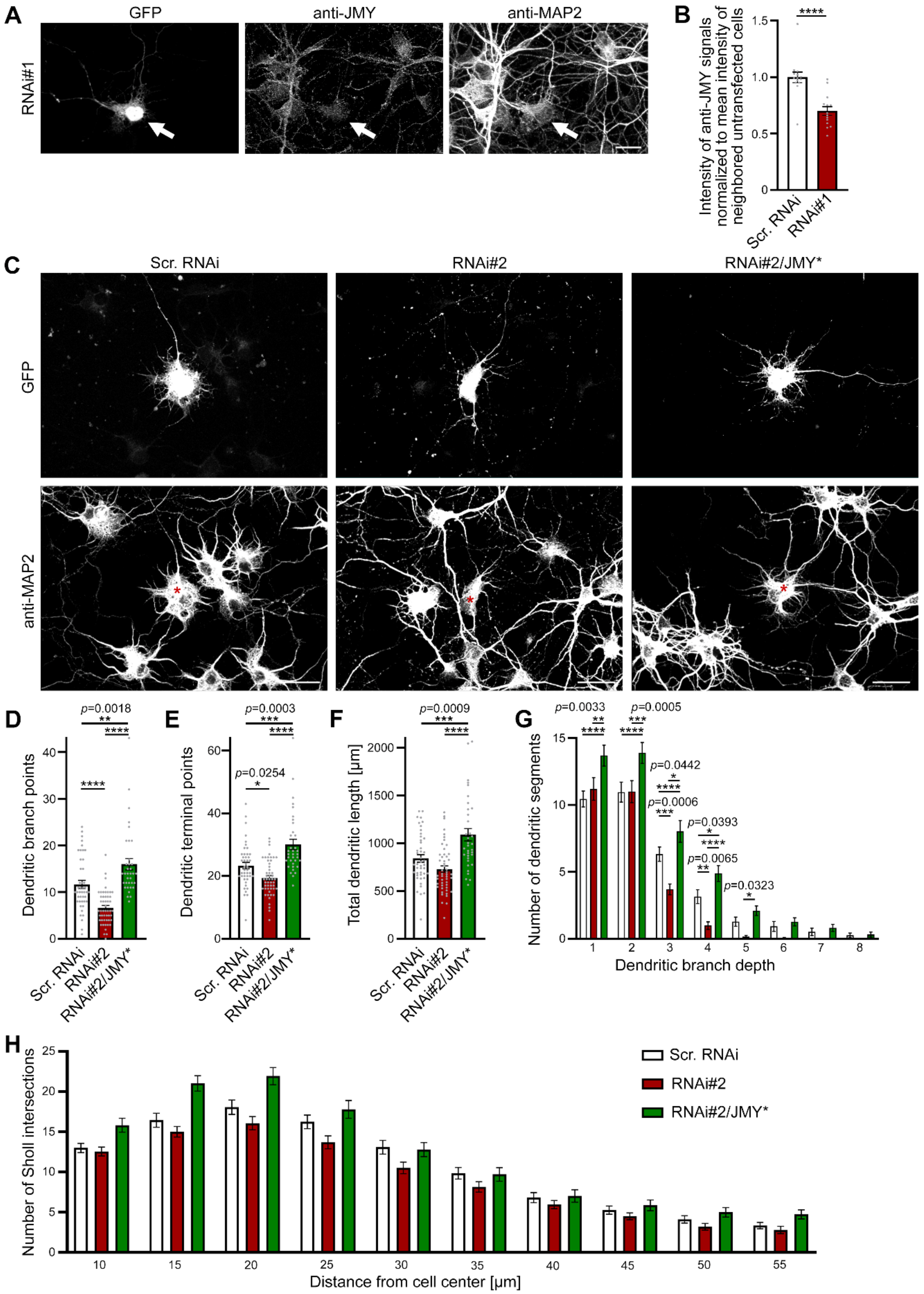


| Distance from cell center [µm] | 10 | 15 | 20 | 25 | 30 | 35 | 40 | 45 | 50 | 55 |
|---|---|---|---|---|---|---|---|---|---|---|
| Scr. RNAi vs. RNAi#2 | ns | ns | ns | * | * | ns | ns | ns | ns | ns |
| Scr. RNAi vs. RNAi#2/JMY* | * | **** | *** | ns | ns | ns | ns | ns | ns | ns |
| RNAi#2 vs. RNAi#2/JMY* | ** | **** | **** | *** | ns | ns | ns | ns | ns | ns |

**Supplementary Figure 2. JMY deficiency leads to reduced dendritic arbor complexity.**

(**A**) MIP of anti-JMY- and anti-MAP2-immunostained primary hippocampal neurons (DIV6) transfected with a plasmid encoding for JMY RNAi#1 and a GFP reporter at DIV4 (arrow marks a JMY RNAi#1-transfected neuron with reduced anti-JMY immunolabeling). Bar, 20 µm. (**B**) Quantification of JMY knockdown in JMY RNAi#1-transfected neurons based on anti-JMY immunofluorescence detections, which were each analyzed in relation to that of a neighbored, untransfected control cell. (**C-H**) MIPs of anti-MAP2-immunostained and GFP-coexpressing primary hippocampal neurons (**C**) and quantitative determinations of specific defects in dendritic arborization caused by the second JMY RNAi (RNAi#2) and rescue thereof with an RNAi-resistent version of JMY, JMY*, addressing dendritic branch points (**D**), dendritic terminal points (**E**), total dendritic length (**F**), as well as by conducting dendritic branch depth analyses (**G**) and Sholl analyses (**H**). Bars, 30 µm. Transfected cells are marked by asterisks. Data, mean±SEM shown as bar/dot plots (**B,D-F**) and bar plot (**G,H**), respectively. (**B**) Scr. RNAi and RNAi#1, n=14 somatic ROIs and neurons each (each in relation to a neighbored control neuron) from 4 independently immunostained coverslips each (from 2 independent assays and neuronal preparations). (**D-H**) Scr. RNAi, n=44 (same data as in Fig. 2); RNAi#2, n=50 from 3 independent assays; RNAi#2/JMY*, n=35 cells from 2 independent neuronal preparations. Numerical data is provided in Supplementary Data 1. Statistical significances, Mann-Witney U test (**B**), One-way ANOVA/Tukey′s post-test (**D-F**) and Two-way ANOVA/Bonferroni′s test (**G,H**); ns, not significant (table only), *$p$<0.05, **$p$<0.01, ***$p$<0.001, ****$p$<0.0001,. For exact $p$ values in **B,D-G** see figure panels. Note that ****$p$<0.0001 are too small values to be reported by the software used.

## Supplementary Figure S3

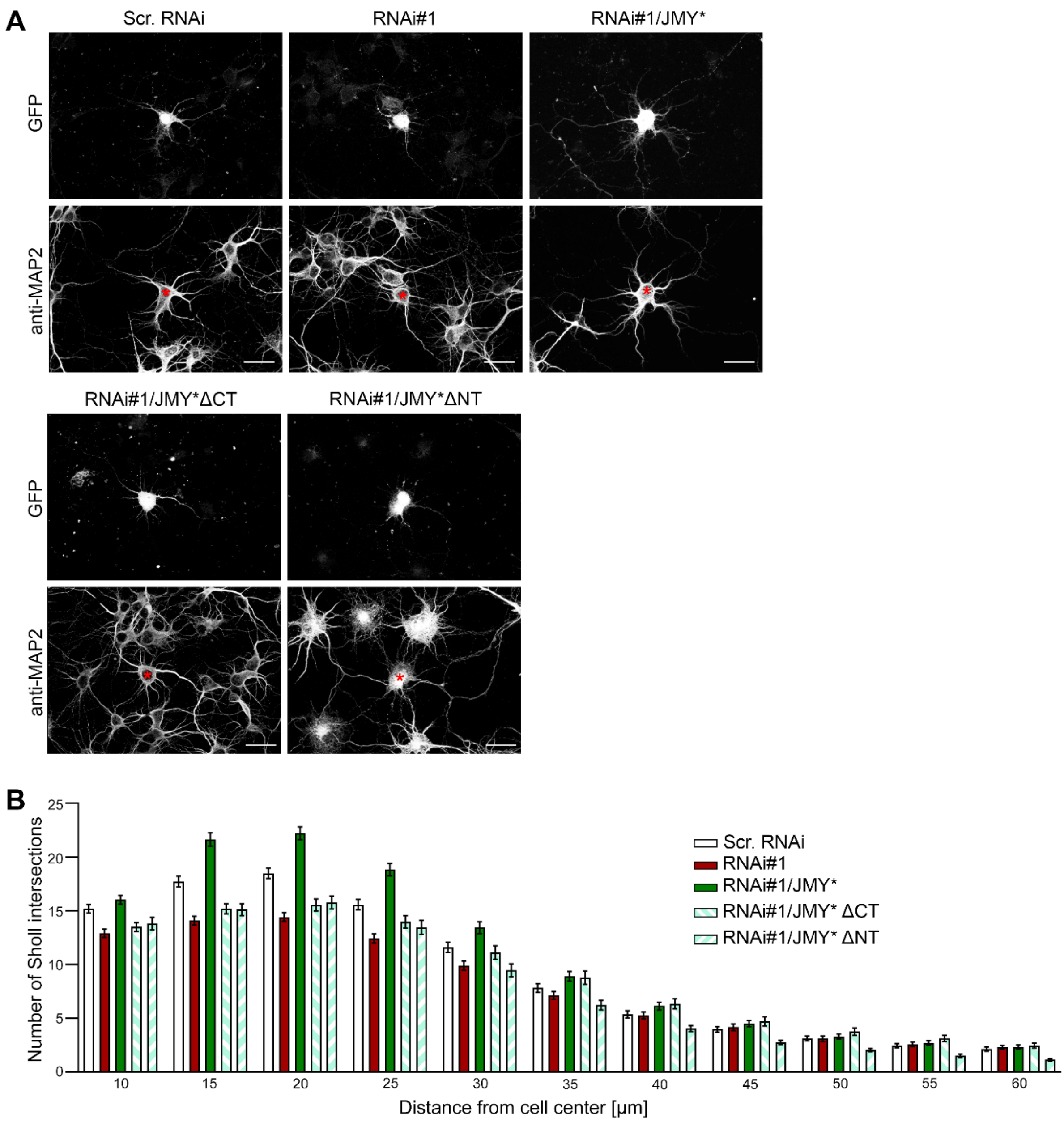


| **Distance from cell center [µm]** | **10** | **15** | **20** | **25** | **30** | **35** | **40** | **45** | **50** | **55** | **60** |
|---|---|---|---|---|---|---|---|---|---|---|---|
| Scr. RNAi vs. RNAi#1 | ns | ** | *** | ** | ns | ns | ns | ns | ns | ns | ns |
| Scr. RNAi vs. RNAi#1/JMY* | ns | **** | **** | **** | * | ns | ns | ns | ns | ns | ns |
| Scr. RNAi vs. RNAi#1/JMY* ΔCT | ns | * | *** | * | ns | ns | ns | ns | ns | ns | ns |
| Scr. RNAi vs. RNAi#1/JMY* ΔNT | ns | * | ** | ** | *** | ** | ns | ns | ns | ns | ns |
| RNAi#1 vs. RNAi#1/JMY* | * | **** | **** | **** | **** | ns | ns | ns | ns | ns | ns |
| RNAi#1 vs. RNAi#1/JMY* ΔCT | ns | ns | ns | ns | ns | ns | ns | ns | ns | ns | ns |
| RNAi#1 vs. RNAi#1/JMY* ΔNT | ns | ns | ns | ns | ns | ns | ns | ns | ns | ns | ns |
| RNAi#1/JMY* vs. RNAi#1/JMY* ΔCT | * | **** | **** | **** | **** | ns | ns | ns | ns | ns | ns |
| RNAi#1/JMY* vs. RNAi#1/JMY* ΔNT | ns | **** | **** | **** | **** | **** | ** | * | ns | ns | ns |
| RNAi#1/JMY* ΔCT vs. RNAi#1/JMY* ΔNT | ns | ns | ns | ns | ns | ** | ** | * | ns | ns | ns |

**Supplementary Figure 3. Both N and C terminal functions of JMY are required for its role in dendritic arborization.**

(**A**) MIPs of anti-MAP staining and GPF signals (reporter) of primary hippocampal neurons transfected with plasmids driving the expression of scr. RNAi, RNAi#1, RNAi#1/JMY*, RNAi#1/JMY* ΔNT and RNAi#1/JMY* ΔCT, respectively, at DIV4 and fixed 30 h thereafter. Asterisks mark transfected neurons. Bars, 30 µm. (**B**) Sholl analyses of neurons expressing scr. RNAi, RNAi#1 against JMY alone or together with RNAi-resistant JMY*, JMY* ΔCT or JMY* ΔNT as rescue attempts. Data, mean±SEM shown as bar plot. Scr. RNAi, RNAi#1, and RNAi#1/JMY*, n=75 neurons (partially included in Fig. 2, too); JMY* ΔCT and JMY* ΔNT, n=45 neurons each from 2-5 independent neuronal preparations. Numerical data are provided in Supplementary Data 1. Statistical significances, Two-way ANOVA/Bonferroni's test. ns, not significant, *$p$<0.05, **$p$<0.01, ***$p$<0.001, ****$p$<0.0001.

## Supplementary Figure S4

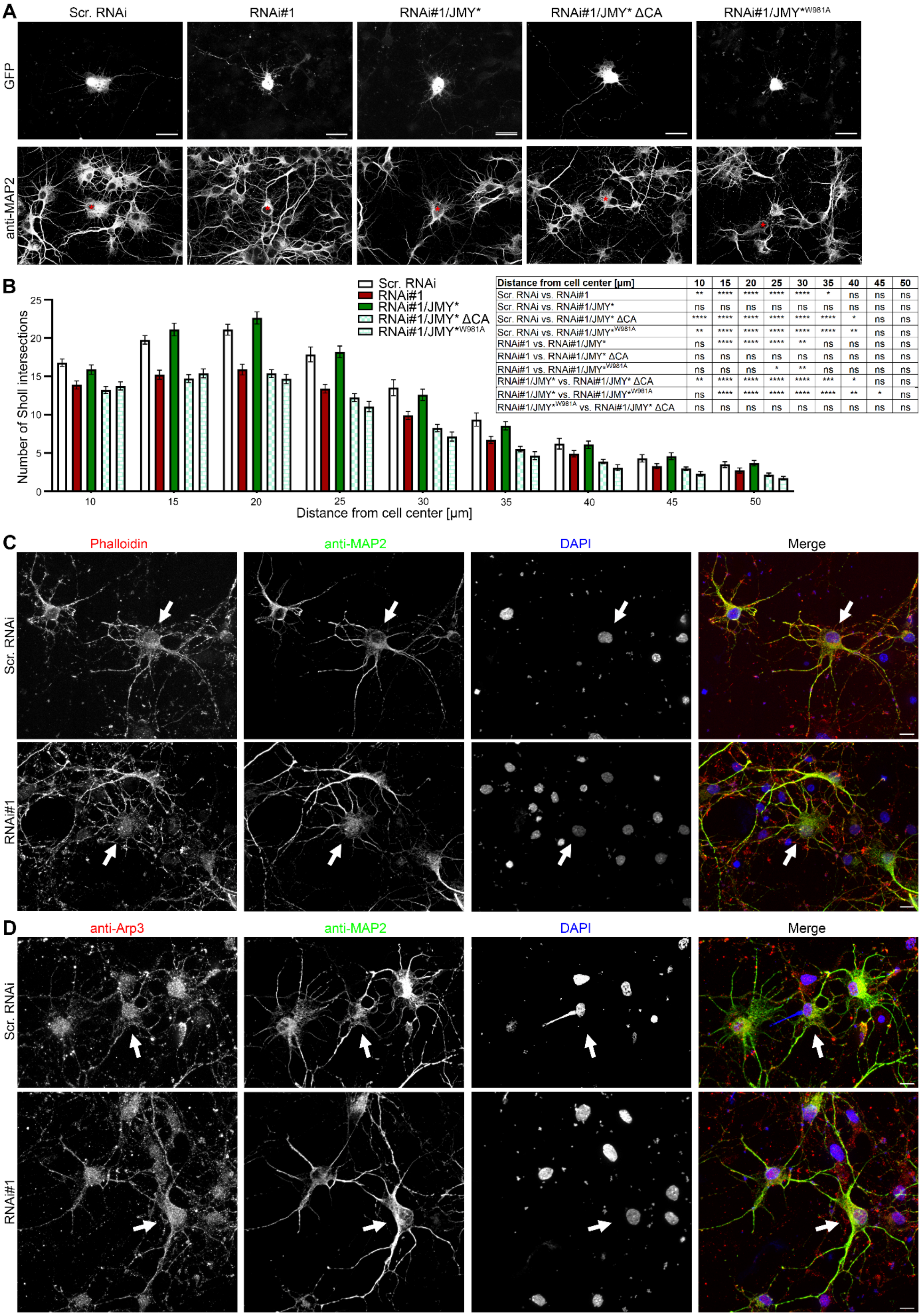


| Distance from cell center [μm] | 10 | 15 | 20 | 25 | 30 | 35 | 40 | 45 | 50 |
|---|---|---|---|---|---|---|---|---|---|
| Scr. RNAi vs. RNAi#1 | ** | **** | **** | **** | **** | * | ns | ns | ns |
| Scr. RNAi vs. RNAi#1/JMY* | ns | ns | ns | ns | ns | ns | ns | ns | ns |
| Scr. RNAi vs. RNAi#1/JMY* ΔCA | **** | **** | **** | **** | **** | **** | * | ns | ns |
| Scr. RNAi vs. RNAi#1/JMY*$^{W981A}$ | ** | **** | **** | **** | **** | **** | ** | ns | ns |
| RNAi#1 vs. RNAi#1/JMY* | ns | **** | **** | **** | ** | ns | ns | ns | ns |
| RNAi#1 vs. RNAi#1/JMY* ΔCA | ns | ns | ns | ns | ns | ns | ns | ns | ns |
| RNAi#1 vs. RNAi#1/JMY*$^{W981A}$ | ns | ns | ns | * | ** | ns | ns | ns | ns |
| RNAi#1/JMY* vs. RNAi#1/JMY* ΔCA | ** | **** | **** | **** | **** | *** | * | ns | ns |
| RNAi#1/JMY* vs. RNAi#1/JMY*$^{W981A}$ | ns | **** | **** | **** | **** | **** | ** | * | ns |
| RNAi#1/JMY*$^{W981A}$ vs. RNAi#1/JMY* ΔCA | ns | ns | ns | ns | ns | ns | ns | ns | ns |

**Supplementary Figure 4. Arp2/3 complex activation and binding by JMY is essential for dendritic arbor formation of developing neurons.**

(**A**) MIPs of anti-MAP2-immunostained and of GFP reporter-expressing primary hippocampal neurons that were transfected at DIV4 as indicated (transfected neurons are marked by red asterisks) and fixed 30 h after transfection. (**B**) Sholl analyses showing that the reduction in Sholl intersections caused by JMY RNAi#1 in comparison to scr. RNAi can be rescued by coexpression of JMY* but not by JMY* ΔCA or JMY*$^{W981A}$. (**C,D**) Phalloidin (**C**) and anti-Arp3 immunolabeling (**D**) together with anti-MAP2 and DAPI-labeled primary hippocampal neurons transfected with either scr. RNAi or RNAi#1 at DIV4 and fixed 30 h later. Note that no obvious changes of F-actin (**C**) or Arp2/3 complex localization (**D**) were observed in the scr. RNAi and JMY RNAi#1-expressing neurons (white arrows). Bars, 30 µm (**A**) and 10 µm (**C,D**), respectively. Data, mean±SEM shown as bar plot. Scr. RNAi, RNAi#1 and RNAi#1/JMY*, n=75 cells (part of the data were already included in Fig. 2F); RNAi#1/JMY* ΔCA, n=45; RNAi#1/JMY*$^{W981A}$, n=30 neurons from 2-5 independent assays. Numerical data are provided in Supplementary Data 1. Statistical significances, Two-way ANOVA/Bonferroni′s test (**B**). ns, not significant, *$p$<0.05, **$p$<0.01, ***$p$<0.001, ****$p$<0.0001.

# Supplementary Figure S5

A

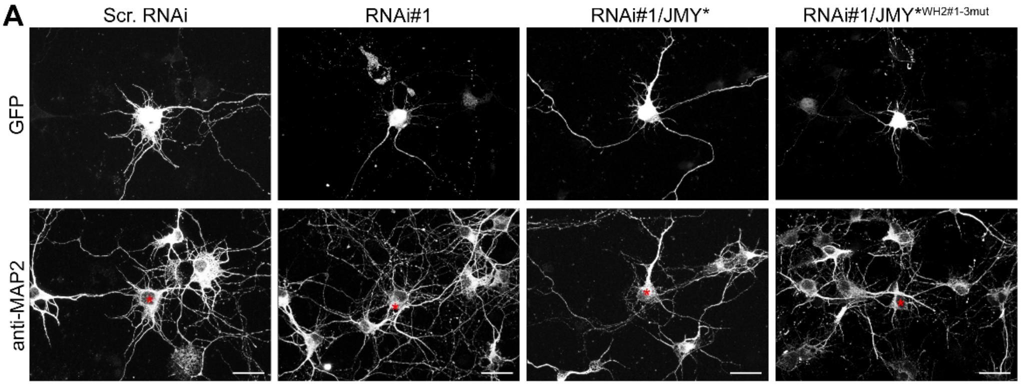


B

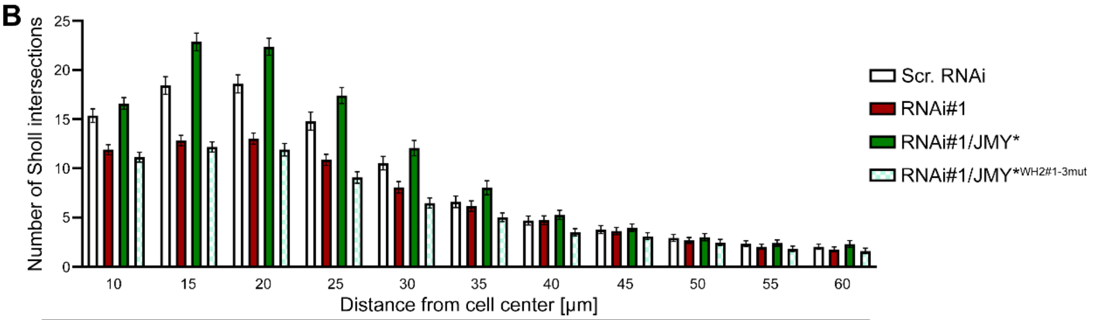


| Distamce from cell center [µm] | 10 | 15 | 20 | 25 | 30 | 35 | 40 | 45 | 50 | 55 | 60 |
|---|---|---|---|---|---|---|---|---|---|---|---|
| Scr. RNAi vs. RNAi#1 | **** | **** | **** | **** | ** | ns | ns | ns | ns | ns | ns |
| Scr. RNAi vs. RNAi#1/JMY* | ns | **** | **** | ** | ns | ns | ns | ns | ns | ns | ns |
| Scr. RNAi vs. RNAi#1/JMY*$^{WH2\#1\text{-}3mut}$ | **** | **** | **** | **** | **** | ns | ns | ns | ns | ns | ns |
| RNAi#1 vs. RNAi#1/JMY* | **** | **** | **** | **** | **** | ns | ns | ns | ns | ns | ns |
| RNAi#1 vs. RNAi#1/JMY*$^{WH2\#1\text{-}3mut}$ | ns | ns | ns | ns | ns | ns | ns | ns | ns | ns | ns |
| RNAi#1/JMY* vs. RNAi#1/JMY*$^{WH2\#1\text{-}3mut}$ | **** | **** | **** | **** | **** | *** | ns | ns | ns | ns | ns |

C

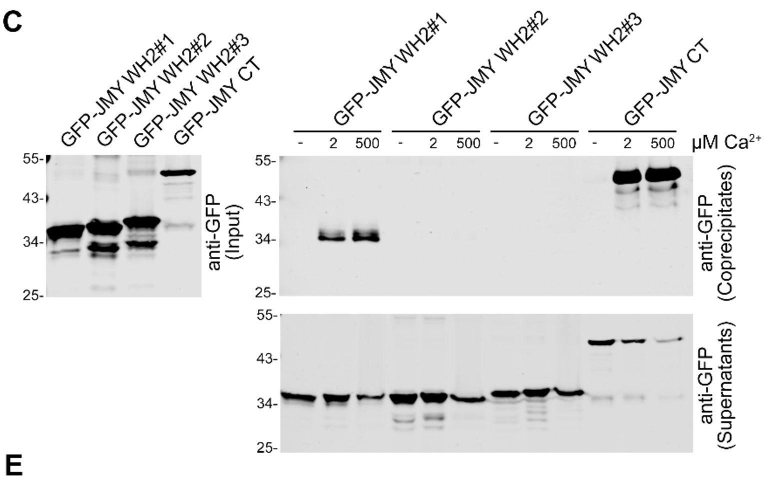


D

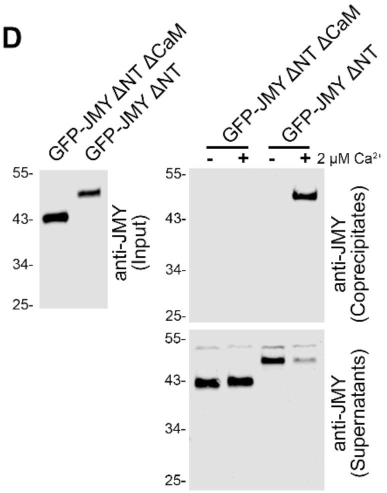


E

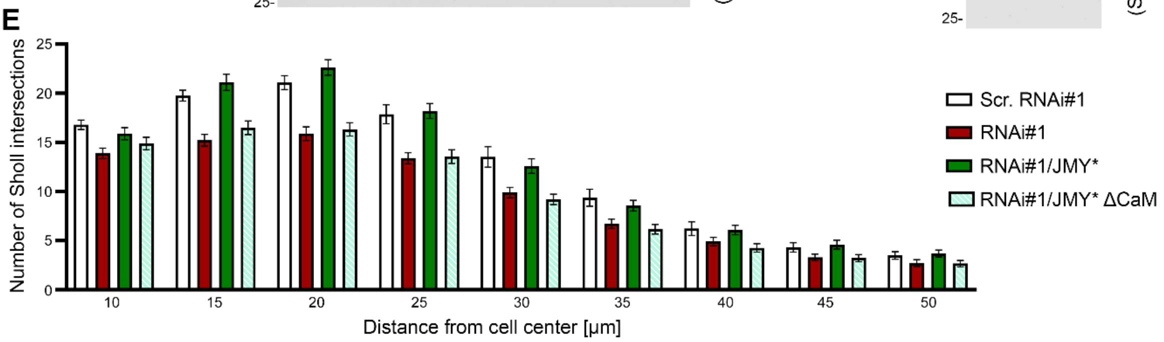


| Distance from cell center [µm] | 10 | 15 | 20 | 25 | 30 | 35 | 40 | 45 | 50 |
|---|---|---|---|---|---|---|---|---|---|
| Scr. RNAi vs. RNAi#1 | ** | **** | **** | **** | *** | * | ns | ns | ns |
| Scr. RNAi vs. RNAi#1/JMY* | ns | ns | ns | ns | ns | ns | ns | ns | ns |
| Scr. RNAi vs. RNAi#1/JMY* ΔCaM | ns | *** | **** | **** | **** | ** | ns | ns | ns |
| RNAi#1 vs. RNAi#1/JMY* | ns | **** | **** | **** | * | ns | ns | ns | ns |
| RNAi#1 vs. RNAi#1/JMY* ΔCaM | ns | ns | ns | ns | ns | ns | ns | ns | ns |
| RNAi#1/JMY* vs. RNAi#1/JMY* ΔCaM | ns | **** | **** | **** | *** | * | ns | ns | ns |

**Supplementary Figure 5. JMY's functions in dendritogenesis rely on actin binding and on a CaM binding region comprising the first WH2 domain of JMY.**

(**A,B**) Representative images (MIPs) of anti-MAP2-immunostained and GFP-expressing (reporter) primary hippocampal neurons transfected at DIV4 with either scr. RNAi, RNAi#1 against JMY, RNAi#1 together with RNAi insensitive JMY* or JMY*$^{WH2\#1\text{-}3mut}$ (transfected cells marked by asterisks) and fixed 30 h after transfection (**A**) and Sholl analyses thereof (**B**) corresponding to the analyses shown in Fig. 5A-D. Bars, 30 µm. (**C**) Immunoblots of lysates of L929 cells expressing GFP-JMY WH2#1, GFP-JMY WH2#2, GFP-JMY WH2#3 and GFP-JMY CT and immunblots of supernatants and coprecipitates with CaM under 2 µM $Ca^{2+}$ and 500 µM $Ca^{2}$. GFP-JMY CT and GFP-JMY WH2#1 were coprecipitated with immobilized CaM matrix under both $Ca^{2+}$ concentrations, but not under the $Ca^{2+}$ free (-) condition, whereas, irrespective of the presence or absence of $Ca^{2+}$, GFP-JMY WH2#2 and GFP-JMY WH2#3 showed no coprecipitation. (**D**) Immunoblots of coprecipitation analyses with GFP- JMY ΔNT and a JMY deletion mutant lacking the CaM binding region (GFP-JMY ΔNT ΔCaM) expressed in L929 cells and immobilized CaM. Note that GFP-JMY ΔNT readily coprecipitated with immobilized CaM but GFP-JMY ΔNT ΔCaM was unable to associate with CaM. (**E**) Sholl analyses of primary hippocampal neurons accompanying the data shown in Fig. 5H-J. Neurons were transfected with scr. RNAi, RNAi#1 against JMY, RNAi#1 coexpressing RNAi insensitive JMY* and JMY* ΔCaM, respectively, at DIV4 and fixed 30 hours later. GFP was expressed as reporter via an inserted IRES site. Note that JMY* but not JMY* ΔCaM was able to rescue the dendritic complexity phenotype caused by JMY RNAi#1. Data, mean±SEM shown as bar plots. n=30 cells for each condition in **B** and **E** (scr. RNAi control, RNAi#1 and RNAi#1/JMY* data (**E**) were also included in Fig. S4B) from 2 independent assays. Numerical data and images of uncropped and unedited blots are provided in Supplementary Data 1 and Supplementary Data 2, respectively. Statistical significances, Two-way ANOVA/Bonferroni′s test (**B,E**). ns, not significant, *$p<0.05$, **$p<0.01$, ***$p<0.001$, ****$p<0.0001$.

## Supplementary Figure S6

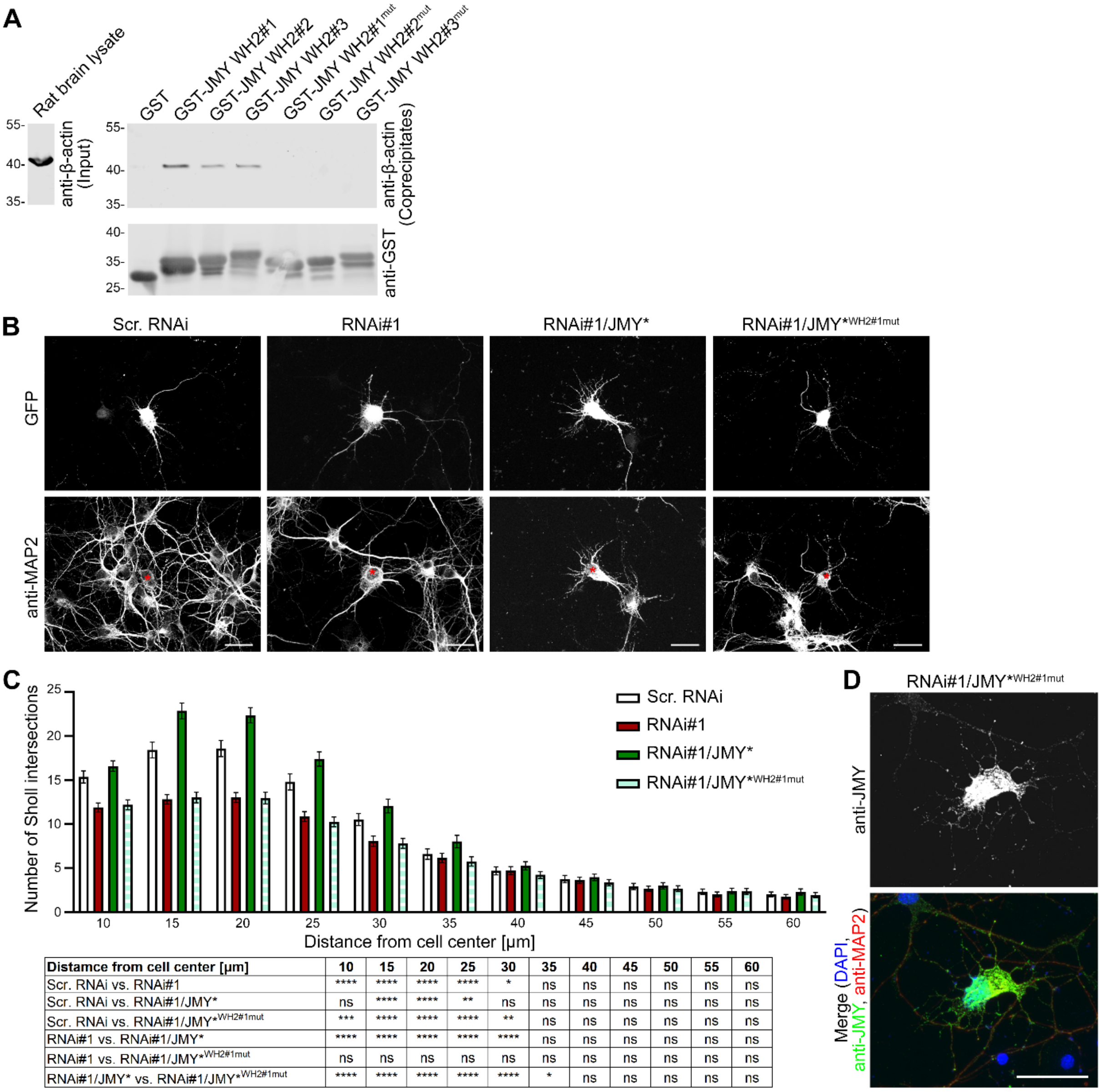


| Distamce from cell center [μm] | 10 | 15 | 20 | 25 | 30 | 35 | 40 | 45 | 50 | 55 | 60 |
|---|---|---|---|---|---|---|---|---|---|---|---|
| Scr. RNAi vs. RNAi#1 | **** | **** | **** | **** | * | ns | ns | ns | ns | ns | ns |
| Scr. RNAi vs. RNAi#1/JMY* | ns | **** | **** | ** | ns | ns | ns | ns | ns | ns | ns |
| Scr. RNAi vs. RNAi#1/JMY*WH2#1mut | *** | **** | **** | **** | ** | ns | ns | ns | ns | ns | ns |
| RNAi#1 vs. RNAi#1/JMY* | **** | **** | **** | **** | **** | ns | ns | ns | ns | ns | ns |
| RNAi#1 vs. RNAi#1/JMY*WH2#1mut | ns | ns | ns | ns | ns | ns | ns | ns | ns | ns | ns |
| RNAi#1/JMY* vs. RNAi#1/JMY*WH2#1mut | **** | **** | **** | **** | **** | * | ns | ns | ns | ns | ns |

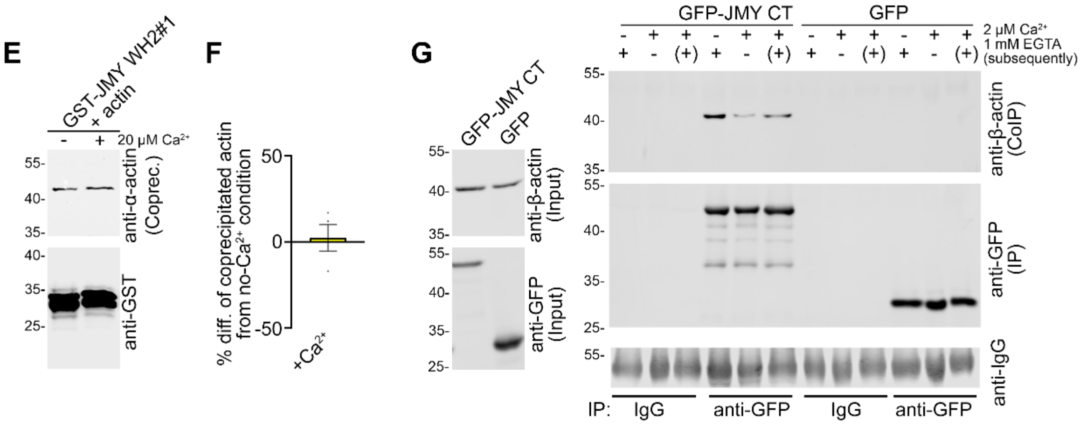

**Supplementary Figure 6. JMY's first WH2 domain interacts with actin most strongly, is key in JMY-mediated dendritic arbor formation and is controlled in a reversible $Ca^{2+}$-dependent manner.**

(**A**) Immunoblot analyses of coprecipitations of endogenous actin from rat brain lysates using immobilized individual GST-JMY WH2 domains and corresponding point mutants thereof ($WH2^{mut}$). Note that all three individual GST-JMY WH2 domains but not their corresponding mutants were able to coprecipitate endogenous actin from rat brain lysate. The integrity of the GST-fusion proteins was confirmed with anti-GST immunoblot analyses of the eluates (lower panel). (**B**) MIPs of anti-MAP stainings of primary hippocampal neurons transfected as indicated and shown in Fig. 6E (transfection at DIV4 and fixation 30 h later) complemented with images of the GPF reporter in the transfected neurons (marked by asterisks in the anti-MAP2 images). (**C**) Sholl analyses of developing primary hippocampal neurons expressing scr. RNAi, RNAi#1, RNAi#1/JMY* and $JMY^{*WH2\#1mut}$, respectively. (**D**) MIPs of hippocampal neurons transfected and fixed under the same conditions as for the morphometric analyses (**B,C**) with additional anti-JMY immunostaining and DAPI staining showing that $JMY^{*WH2\#1mut}$ not able to rescue was expressed and available in soma and dendrites. Bars, 30 μm. (**E,F**) Immunoblottings (**E**) and quantitative evaluations using fluorescence-based Western blots (**F**) of in vitro reconstitution of JMY WH2#1/actin complexes with purified proteins (GST-JMY WH2#1 immobilized) demonstrating that actin binding of JMY WH2#1 is not altered when $Ca^{2+}$ and no-$Ca^{2+}$ conditions are compared. (**G**) Example of the immunoblot analyses of coprecipitates of endogenous actin with GFP-JMY CT from L929 cell lysates under three different $Ca^{2+}$ conditions used for the quantitative analyses shown in Fig. 6. Note that the impairment of the actin binding ability of GFP-JMY CT in the presence of 2 μM $Ca^{2+}$ was partially reversible upon subsequent chelation of $Ca^{2+}$ with EGTA. Data, mean±SEM shown as bar plot (**C**) and bar/dot plot (**F**), respectively. Scr. RNAi, RNAi#1, RNAi#1/JMY* and RNAi#1/$JMY^{*WH2\#1mut}$ n=30 neurons from 2 independent neuronal preparations (data partially

also shown in Fig. S5B). Numerical data and images of uncropped and unedited blots are provided in Supplementary Data 1 and Supplementary Data 2, respectively. Statistical differences, Two-way ANOVA/Bonferroni′s test (**C**) and Welch′s t test (**F**), respectively. ns, not significant, *$p$<0.05, **$p$<0.01, ***$p$<0.001, ****$p$<0.0001.

## Supplementary Figure S7

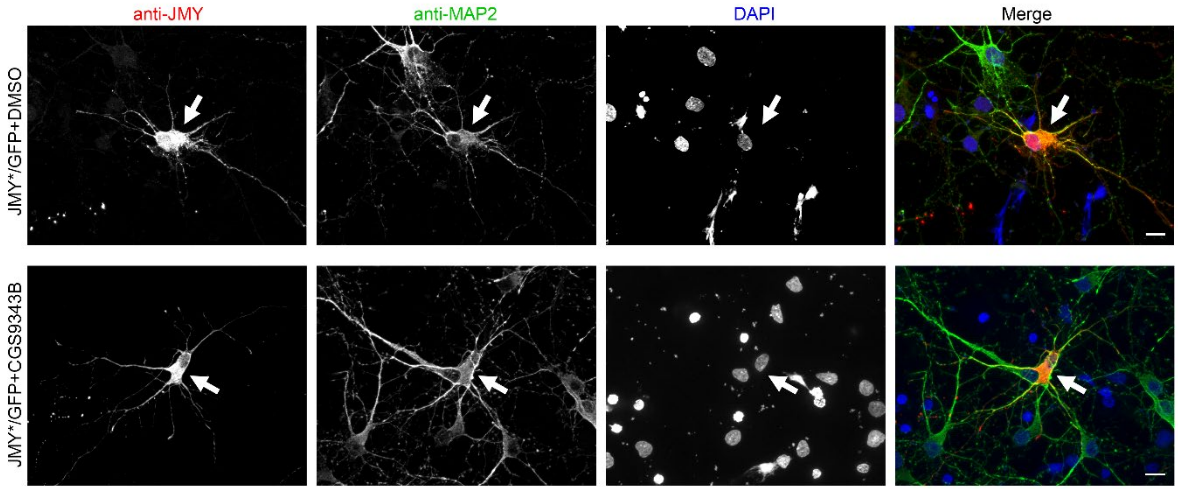


**Supplementary Figure 7. The calmodulin inhibitor CGS9343B does not lead to any obvious changes in JMY localization in developing neurons.**

MIPs of anti-JMY, anti-MAP2 and DAPI (immuno)stainings of primary, JMY*-expressing hippocampal neurons (additional GFP reporter not shown in the 3 color merge for clarity; transfected cells marked with white arrow) not showing any obvious changes of JMY* localization upon application of the calmodulin inhibitor CGS9343B. Transfection at DIV4, fixation 30 h later. Bars, 10 µm.

**Supplementary Table 1. List of primers used**

The sequences and additional information for the primers used in this study can be found in this table. F-primer, forward primer; R-primer, reverse primer.

| **Purpose, position and sequence of primer**<br>**(Mus musculus JMY; GI: 61098108)** | **Restriction site and mutations introduced, respectively** |
|---|---|
| F-primer: aa502-506<br>5′-caaagcttacagggtggta-3′ | HindIII |
| R-primer: aa826-830<br>5′-ttctcgagtcactctgcagtggtgga-3′ | XhoI |
| F-primer: aa 821-825<br>5′-aagtcgacaaggacaatggggcc-3′ | SalI |
| R-primer: aa983-979<br>5′-aacccgggctagttctcccagtctgt-3′ | SmaI |
| F-primer: aa 841-845<br>5′-acgaattcggcaatgagaggagc-3′ | EcoRI |
| R-primer: 883-879<br>5′- gtgtcgacctaactcactctcctcct-3′ | SalI |
| F-primer: aa 874-878<br>5′-acgaattcgtggaagggctgcag-3′ | EcoRI |
| R-primer: aa 914-910<br>5′-gtgtcgacctattcatcaggaaaagg-3′ | SalI |
| F-primer: aa 904-908<br>5′-acgaattcgaacagcggactctg-3′ | EcoRI |
| R-primer: aa 947-943<br>5′-gtgtcgacctatggggacgcttcttt-3′ | SalI |

| | |
|---|---|
| F-primer: aa 710-714<br><br>5′-tagctagcgccaccatgagcacagcaagccct-3′ | NheI |
| R-primer: aa 983-979<br><br>5′-aactcgagctagttctcccagtctgt-3′ | XhoI |
| F-primer: aa 853-874<br><br>5′-cccgcagcacacgcagcagatagcagccagctggtcagcgcacggaaaaaggcaagaaagactgtg-3′ | WH2#1 mut<br><br>L857A, F858A, L870A |
| F-primer: aa 886-904<br><br>5′-atggatgaagcagcagcatccttgaagcgtggtagctttcatgcaaaaaaggttgaa-3′ | WH2#2 mut<br><br>V889A, L890A, L900A |
| F-primer: aa 917-934<br><br>5′-agtaataatgcagcagcgcagataaggaaaggggtaaaggcaaagaaggttcag-3′ | WH2#3 mut<br><br>I920A, L921A, L930A |
| F-primer for new multiple cloning site insertion into IRES-GFP vector<br><br>5′-ctagcgctgcccgggcactagtctcgagatcgatcccgggtctagagc-3′ | NheI, AfeI, SmaI, SrfI, SpeI,<br><br>XhoI, ClaI, SmaI, XbaI, NotI |
| R-primer for multiple cloning site insertion into IRES-GFP vector<br><br>5′-ggccgctctagacccgggatcgatctcgagactagtgcccgggcagcg-3′ | NheI, AfeI, SmaI, SrfI, SpeI,<br><br>XhoI, ClaI, SmaI, XbaI, NotI |
| F-primer: nt 69-89 (aa 23-30), RNAi#1<br><br>5′-gatccgcgcgagaagcacaagtttgtttgatatccgacaaacttgtgcttctcgcgctttttta-3′ | BamHI |
| R-primer: nt 69-89 (aa 23-30), RNAi#1<br><br>5′-agcttaaaaaagcgcgagaagcacaagtttgtcggatatcaaacaaacttgtgcttctcgcgcg-3′ | HindIII |
| F-primer: nt 78-98 (aa 26-33), RNAi#2<br><br>5′-gatccgcacaagtttgtgttcattgtttgatatccgacaatgaacacaaacttgtgctttttta-3′ | BamHI |
| R-primer: nt 78-98 (aa 26-33), RNAi#2<br><br>5′-agcttaaaaaagcacaagtttgtgttcattgtcggatatcaaacaatgaacacaaacttgtgcg-3′ | HindIII |
| F-primer nt 57-108 (aa 19-36) for silent mutation of codons<br><br>5′-cgtattcgacgaaagggaaaagcataaattcgtatttatcgtggcctggaac-3′ | RNAi#1 & RNAi#2<br><br>insensitive mutation |
| R-primer nt 57-108 (aa 19-36) for silent mutation of codons<br><br>5′-gttccaggccacgataaatacgaatttatgcttttccctttcgtcgaatacg-3′ | RNAi#1 & RNAi#2<br><br>insensitive mutation |
| R-primer: aa 938-934 | XhoI |

| | |
|---|---|
| 5′-atctcgagtcacaaaacttccttctg-3′ | |
| R-primer: aa 983-977 W981A<br><br>5′-cagaattcctactagttctctgcgtctgtgca-3′ | EcoRI |

**Supplementary Data 1. Numerical data underlying quantitative analyses.**

Compilation of numerical data underlying all of the quantitative figure panels shown in this study (see separate file).

**Supplementary Data 2. Compilation of all uncropped and unedited blots shown in the main and supplementary figures.**

The compilation shows the uncropped and unedited blots presented in both the main and the supplementary figures (labeling according to the figures). Additional dashed lines indicate where the blot images were cropped in order to be integrated into the respective figure (see separate file).
