# Supplementary material for: JMY powers dendritogenesis and is regulated by CaM revealing a general, critical principle in neuromorphogenesis
Source: Commun Biol. 2025 May 22;8:784. doi: 10.1038/s42003-025-08208-3 (PMC12098658; doi:10.1038/s42003-025-08208-3)
Supplement: Supplementary file 3 — Description of Additional Supplementary Files [file 42003_2025_8208_MOESM3_ESM.docx]

Description of Additional Supplementary Files

**File name:** Supplementary Data 1

**Description:** Compilation of numerical data underlying all of the quantitative figure panels shown in this study (see separate file).

**File name:** Supplementary Data 2

**Description:** The compilation shows the uncropped and unedited blots presented in both the main and the supplementary figures (labelling according to the figures). Additional dashed lines indicate where the blot images were cut in order to be integrated into the respective figure (see separate file).
