# Supplementary material for: JMY powers dendritogenesis and is regulated by CaM revealing a general, critical principle in neuromorphogenesis
Source: Commun Biol. 2025 May 22;8:784. doi: 10.1038/s42003-025-08208-3 (PMC12098658; doi:10.1038/s42003-025-08208-3)
Supplement: Supplementary file 4 — Supplementary Data 1 [file 42003_2025_8208_MOESM4_ESM.pdf]

Fig 1B

	EGTA	2 µM Ca^{2+}	50 µM Ca^{2+}
	1	16,35	27,02
	1	20,76	29,19
**mean**	1,00	18,56	28,11
**SD**	0,00	3,12	1,53
**n**	2	2	2
**SEM**	0,00	2,21	1,09

Fig 1H
Dendritic branch points

	GFP	JMY*/GFP
	12	8
	6	10
	8	13
	7	16
	15	16
	5	10
	4	20
	14	12
	6	12
	7	16
	7	22
	16	12
	5	25
	17	4
	7	28
	5	11
	10	13
	7	8
	6	14
	7	12
	9	14
	8	13
	6	14
	5	14
	7	23
	7	17
	6	22
	6	12
	6	13
	5	16
**mean**	7,87	14,67
**SD**	3,46	5,29
**n**	30	30
**SEM**	0,63	0,97

Fig 1I
Dendritic terminal points

GFP	JMY*/GFP
28	19
21	23
20	26
16	30
22	26
17	26
14	32
27	29
21	19
21	29
16	37
29	26
18	41
27	19
15	46
20	25
24	25
19	21
17	29
16	26
22	26
27	24
16	23
18	27
23	38
13	39
20	33
17	25
21	34
18	31
20,10	28,47
4,33	6,67
30	30
0,79	1,22

Fig 1J
Total dendritic length [μm]

GFP	JMY*/GFP
873,2	1189,3
878,1	731,3
790,4	1212,2
671,3	1065,8
940,1	762,3
593,9	1161,2
516,8	1453,7
971,4	788,1
1017,0	644,2
783,6	1068,5
690,0	1717,1
987,5	1006,7
907,1	1340,5
1027,2	535,1
622,3	1336,8
757,5	1156,1
1205,6	733,7
830,6	819,8
786,8	1157,2
577,8	791,4
1071,3	848,1
962,0	977,0
501,7	902,6
802,4	1260,0
976,5	1155,6
718,1	1230,6
947,7	839,3
715,1	791,0
949,8	1197,0
696,2	1017,3
825,62	1029,65
173,73	265,10
30	30
31,72	48,40

Fig 1K
Sholl analyses

Sholl intersections	GFP			JMY*/GFP		
	**Mean**	**SEM**	**n**	**Mean**	**SEM**	**n**
**10**	12,6	0,54	30	15,5	0,78	30
**15**	14,63	0,56	30	20,5	1,01	30
**20**	16,63	0,64	30	22,27	1,06	30
**25**	15,33	0,68	30	17,93	1,03	30
**30**	11,93	0,8	30	12,9	0,84	30
**35**	9,2	0,75	30	9,37	0,64	30
**40**	6,97	0,68	30	6,23	0,49	30
**45**	5,47	0,51	30	4,97	0,44	30
**50**	4,37	0,39	30	4,03	0,35	30
**55**	3,43	0,3	30	3,47	0,36	30
**60**	3	0,27	30	3,17	0,36	30

Fig 1L
Depth analyses

Depth	GFP			JMY*/GFP		
	**Mean**	**SEM**	**n**	**Mean**	**SEM**	**n**
**1**	12,33	0,54	30	13,73	0,74	30
**2**	10,03	0,47	30	14,67	0,76	30
**3**	3,73	0,49	30	8,63	0,74	30
**4**	1,6	0,47	30	3,8	0,58	30
**5**	0,33	0,17	30	1,73	0,3	30
**6**	0,27	0,16	30	0,8	0,26	30
**7**	0,07	0,07	30	0,13	0,09	30
**8**	0	0	30	0,13	0,13	30

Fig 2B

Dendritic branch points

	Scr. RNAi	RNAi#1	RNAi#1/JMY*
	6	5	16
	10	6	13
	9	7	13
	7	4	13
	11	5	14
	10	7	12
	9	8	12
	8	6	10
	12	4	12
	8	4	15
	9	5	10
	10	6	14
	11	5	9
	10	6	12
	10	4	11
	1	13	15
	4	2	16
	5	2	12
	7	11	6
	8	9	21
	8	0	9
	2	0	12
	9	0	16
	19	1	15
	19	1	8
	12	0	30
	5	0	20
	10	0	10
	21	10	14
	8	8	18
	17	10	13
	20	6	30
	11	9	13
	10	9	18
	11	6	21
	19	8	15
	22	8	20
	12	7	10
	24	9	26
	23	7	30
	15	7	
	17	7	
	19	6	
	15	7	
		5	
		6	
**mean**	11,66	5,57	15,10
**SD**	5,68	3,25	5,85
**n**	44	46	40
**SEM**	0,86	0,48	0,92

Fig 2C

Dendritic terminal points

Scr. RNAi	RNAi#1	RNAi#1/JMY*
22	20	32
20	21	29
25	21	30
17	19	22
29	18	24
19	18	28
24	18	26
25	24	21
27	17	23
21	23	27
26	20	23
20	18	29
27	24	19
23	21	25
23	13	24
6	23	29
15	13	28
20	13	30
14	22	18
19	16	35
21	8	21
18	8	27
20	9	29
43	11	26
29	12	20
22	12	43
16	10	31
21	7	26
37	27	29
14	18	30
26	19	28
33	15	42
20	16	22
21	19	34
16	20	35
39	18	30
30	20	39
20	22	26
27	26	31
31	16	48
25	20	
25	18	
27	17	
26	21	
	13	
	20	
23,39	17,48	28,48
6,85	4,85	6,50
44	46	40
1,03	0,72	1,03

Fig 2D

Total dendritic length [µm]

Scr. RNAi	RNAi#1	RNAi#1/JMY*
743,13	979,64	1035,1
806,5	942,24	791,54
922,64	1088,39	970,82
577,27	736,94	988,49
1335,36	680,1	689,45
671,56	800,6	1161,71
763,88	622,15	923,15
1032,4	1013	751,86
914,78	612,04	817,68
635,49	873,77	826,92
1051,54	802,67	729,26
1213,04	784,38	1005,6
778,25	911,44	712,26
740,21	836,64	990,86
636,33	432,2	1020,79
204,07	832,04	670,29
491,07	745,89	1088,61
898,43	496,09	1006,5
693,62	751,66	609,85
817,05	446,42	1229,37
683,14	499,32	625,8
689,33	293,47	692,07
685,7	302,21	950,73
1224,67	354,49	733,93
1137,76	448,39	920,12
665,11	326,93	1222,61
547,78	455,88	1054,35
846,25	293,73	911,58
1268,9	872,74	903,35
476,79	513,63	942,4
1335,51	576,66	925,53
1099,3	642,66	1480,76
697,74	654,03	685,53
773,81	653,68	1060,63
467,98	830,56	1152,2
1275,19	654,79	979,53
1009,5	676,41	1348,79
738,95	708,62	980,22
780,53	955,69	991,69
1070,75	774,76	1691,25
905,82	505,93	
948,01	628,58	
1180,66	633,81	
630,37	589,16	
	539,24	
	695,28	
842,41	662,37	956,83
260,47	201,71	229,42
44	46	40
39,27	29,74	36,27

Fig 2E

Depth analyses

	Scr. RNAi			RNAi#1			RNAi#1/JMY*		
**Depth**	**Mean**	**SEM**	**n**	**Mean**	**SEM**	**n**	**Mean**	**SEM**	**n**
**1**	10,45	0,59	51	10,72	0,55	46	13,18	0,45	39
**2**	10,96	0,74	51	8,78	0,58	46	14,36	0,63	39
**3**	6,33	0,54	51	3,37	0,43	46	8,03	0,65	39
**4**	3,16	0,52	51	0,91	0,19	46	3,72	0,45	39
**5**	1,29	0,34	51	0,09	0,06	46	1,59	0,35	39
**6**	0,94	0,37	51	0	0	46	0,9	0,27	39
**7**	0,53	0,27	51	0	0	46	0,77	0,24	39
**8**	0,27	0,17	51	0	0	46	0,44	0,19	39

Fig 2F

Sholl analyses

**Sholl**	Scr. RNAi			RNAi#1			RNAi#1/JMY*		
**intersections**	**Mean**	**SEM**	**n**	**Mean**	**SEM**	**n**	**Mean**	**SEM**	**n**
**10**	13	0,57	44	11,2	0,53	46	14,73	0,46	40
**15**	16,45	0,88	44	13,57	0,62	46	19,63	0,79	40
**20**	18,07	0,89	44	14	0,71	46	21,48	0,78	40
**25**	16,25	0,85	44	12,28	0,76	46	17,4	0,82	40
**30**	13,09	0,86	44	9,72	0,7	46	12,63	0,75	40
**35**	9,86	0,72	44	6,72	0,53	46	8,65	0,6	40
**40**	6,82	0,62	44	5,07	0,42	46	5,3	0,42	40
**45**	5,27	0,52	44	4,22	0,42	46	3,85	0,32	40
**50**	4,11	0,46	44	3,26	0,33	46	3,13	0,28	40
**55**	3,34	0,39	44	2,91	0,29	46	3,1	0,33	40

Fig 3C
Dendritic branch points

	Scr. RNAi	RNAi#1	RNAi#1/JMY*	RNAi#1/JMY* ΔCT	RNAi#1/JMY* ΔNT
	6	5	16	12	3
	10	6	13	4	2
	9	7	13	7	8
	7	4	13	5	5
	11	5	14	7	4
	10	7	12	7	5
	9	8	12	5	3
	8	6	10	10	8
	12	4	12	3	5
	8	4	15	7	5
	9	5	10	4	7
	10	6	14	4	5
	11	5	9	8	8
	10	6	12	9	4
	10	4	11	6	8
	5	4	16	7	7
	7	4	12	5	6
	5	4	11	5	3
	4	4	11	4	6
	6	2	12	6	4
	10	4	14	8	6
	3	4	11	8	2
	7	4	14	5	6
	2	5	13	9	4
	7	2	8	8	3
	8	3	11	5	2
	5	7	13	5	5
	6	2	9	7	6
	5	3	16	6	4
	7	3	10	10	4
	8	4	25	3	3
	7	2	13	5	3
	8	4	15	2	4
	8	3	14	2	3
	7	4	12	3	2
	7	4	19	3	3
	7	4	15	3	3
	5	4	14	3	3
	8	3	21	2	3
	5	4	18	4	3
	5	3	22	2	3
	7	6	20	2	2
	7	4	17	2	5
	6	4	20	3	4
	10	5	20	2	1
	7	4	19		
	8	4	10		
	7	1	14		
	6	3	14		
	10	5	13		
	8	3	15		
	6	4	15		
	10	4	13		
	9	1	13		
	7	3	16		
	7	3	10		
	8	3	20		
	6	3	16		
	8	3	25		
	7	3	13		
	5	3	12		
	7	4	10		
	6	3	14		
	6	4	13		
	7	2	15		
	5	3	9		
	6	3	12		
	5	3	15		
	5	3	14		
	4	2	15		
	6	1	16		
	7	3	12		
	6	4	14		
	6	2	17		
	5	2	13		
**mean**	7,09	3,79	14,12	5,27	4,29
**SD**	1,96	1,41	3,52	2,55	1,83
**n**	75	75	75	45	45
**SEM**	0,23	0,16	0,41	0,38	0,27

Fig 3D
Dendritic terminal points

Scr. RNAi	RNAi#1	RNAi#1/JMY*	RNAi#1/JMY* ΔCT	RNAi#1/JMY* ΔNT
22	20	32	22	13
20	21	29	17	14
25	21	30	26	18
17	19	22	14	16
29	18	24	25	16
19	18	28	18	19
24	18	26	22	16
25	24	21	22	16
27	17	23	17	15
21	23	27	24	14
26	20	23	17	19
20	18	29	18	20
27	24	19	23	28
23	21	25	23	18
23	13	24	16	26
19	20	29	15	28
26	21	25	18	19
20	19	24	16	16
14	23	18	18	16
16	18	23	22	14
20	17	33	24	21
17	19	29	24	20
23	15	29	17	19
13	20	23	19	21
20	10	15	21	19
24	14	26	16	17
17	16	21	18	14
21	10	27	23	13
21	24	31	19	16
28	14	20	23	17
21	14	43	20	18
19	16	27	14	12
22	21	29	14	11
27	20	29	14	18
19	15	27	19	14
26	16	33	21	21
25	17	37	20	17
24	12	25	16	21
26	15	33	17	25
22	19	30	18	20
21	16	35	14	17
19	18	38	13	17
23	19	37	14	20
20	16	35	15	15
21	18	37	11	24
29	18	36		
23	13	21		
20	12	29		
25	14	27		
28	19	27		
22	17	32		
25	16	31		
28	13	27		
28	14	25		
22	18	32		
28	15	24		
24	15	35		
19	13	30		
26	17	41		
21	13	25		
13	14	26		
25	14	24		
16	14	30		
18	20	26		
20	8	27		
14	11	20		
22	23	30		
20	13	28		
16	11	27		
13	11	25		
22	10	28		
16	14	23		
18	14	27		
16	19	28		
17	12	25		
21,55	16,56	27,81	18,60	17,96
4,15	3,74	5,29	3,71	3,91
75	75	75	45	45
0,48	0,43	0,61	0,55	0,58

Fig 3E
Total dendritic length [µm]

Scr. RNAi	RNAi#1	RNAi#1/JMY*	RNAi#1/JMY* ΔCT	RNAi#1/JMY* ΔNT
743,13	979,64	1035,1	847,33	412,27
806,5	942,24	791,54	778,51	482,5
922,64	1088,39	970,82	1189,54	681,63
577,27	736,94	988,49	742,84	625,13
1335,36	680,1	689,45	1135,64	555,21
671,56	800,6	1161,71	779,37	806,38
763,88	622,15	923,15	1084,28	558,4
1032,4	1013	751,86	1057,22	479,09
914,78	612,04	817,68	791,61	513,8
635,49	873,77	826,92	821,42	483,43
1051,54	802,67	729,26	641,73	715,9
1213,04	784,38	1005,6	733,24	701,89
778,25	911,44	712,26	957,07	1021,64
740,21	836,64	990,86	924,44	675,2
636,33	432,2	1020,79	621,58	993,58
667,47	747,67	951,22	559,94	1076,17
945,83	1144,55	878,18	909,81	676,91
721,45	766,42	751,44	545,03	570,16
685,51	1218,86	678,85	911,07	563,8
693,28	690,5	707,98	997,44	588,99
858,96	778,37	1201,01	852,72	682,48
605,05	707,34	968,99	880,21	838,38
825,17	692,33	924,2	702,2	662,97
532,83	921,26	630,14	819,18	770,67
963,19	423,23	674,42	933	745,95
1100,85	526,65	948	571,64	552,63
708,75	622,12	691,41	768,27	608,61
648,15	508,71	791,24	959,48	490,41
857,89	940,52	925,58	851,71	645,51
989,81	472,08	627,07	926,83	576,81
708,65	725,1	1403,76	969,23	648,39
875,85	621,14	730,9	530,77	433,38
699,23	893,94	927,54	701,26	353,12
897,25	934,46	894,22	486,29	598,9
726,76	597,68	922,07	1025,64	590,75
1021,51	775,57	1135,91	998,76	839,55
873,58	753,69	1401,01	694,61	582,71
809,96	569,62	801,37	573,17	749,76
1097,11	591,08	1002,54	501,04	785,26
995,03	873,48	997,61	587,58	669,23
708,88	697,39	1290,65	499,32	606,48
767,34	785,19	1391,38	461,93	661,35
935,46	665,4	1370,64	510,19	590,54
760,84	773,02	1236,07	543,98	474,31
763,78	580,73	1171,47	437,44	998,64
1009,13	649,77	1043,54		
891,87	608,52	759,91		
755,19	693,08	889,09		
996,69	581,91	1126,44		
879,01	920,79	1096,42		
1097,69	760,83	1007,26		
959	773,29	1080,06		
880,59	436,85	868,23		
1004,95	584,94	866,33		
980,49	773,85	1061,03		
988,81	669,46	908,26		
911,11	705,17	1229,39		
763,13	498,2	755,82		
818,19	784,27	1258,44		
897,31	645,94	954,56		
424,42	746,03	716,7		
837,76	526,79	599,17		
407,62	572,05	1061,87		
486,65	901,04	679,51		
693,08	329,01	814,53		
417,23	466,83	539,92		
737,36	675,67	833,59		
614,02	440,1	807,28		
676,27	414,02	769,09		
411,16	373,02	828,59		
824,98	299,77	957,83		
571,74	492,1	775,12		
572,35	528,78	702,24		
518,53	757,92	808,6		
816,26	391,04	762,19		
801,47	694,87	920,04	773,68	651,97
189,15	190,21	203,48	201,77	160,81
75	75	75	45	45
21,84	21,96	23,50	30,08	23,97

Fig 4B

Dendritic branch points

	GFP			JMY*/GFP		
	**Mean**	**SEM**	**n**	**Mean**	**SEM**	**n**
**DMSO**	5,93	0,18	40	10,35	0,36	40
**CK666**	3,5	0,28	30	3,13	0,21	30

Fig 4C

Dendritic terminal points

	GFP			JMY*/GFP		
	**Mean**	**SEM**	**n**	**Mean**	**SEM**	**n**
**DMSO**	17,73	0,56	40	23,58	0,55	40
**CK666**	16,47	0,8	30	15	0,66	30

Fig 4D

Total dendritic length [µm]

	GFP			JMY*/GFP		
	**Mean**	**SEM**	**n**	**Mean**	**SEM**	**n**
**DMSO**	623,46	25,14	40	716,6	22,13	40
**CK666**	594,68	31,89	30	537,25	28,75	30

Fig. 4E

Sholl analyses

**Sholl**	GFP+DMSO			JMY*/GFP+DMSO			GFP+CK666			JMY*/GFP+CK666		
**intersections**	**Mean**	**SEM**	**n**	**Mean**	**SEM**	**n**	**Mean**	**SEM**	**n**	**Mean**	**SEM**	**n**
**10**	12,8	0,52	40	15,75	0,6	40	13,4	0,63	30	12,47	0,51	30
**15**	15,38	0,56	40	20,25	0,77	40	14,73	0,73	30	13,83	0,59	30
**20**	15,23	0,61	40	18,88	0,68	40	14,27	0,79	30	13,17	0,7	30
**25**	12,45	0,69	40	12,83	0,62	40	11,27	0,82	30	10,33	0,69	30
**30**	9,05	0,66	40	7,85	0,52	40	7,53	0,73	30	6,8	0,62	30
**35**	5,93	0,53	40	4,83	0,42	40	5,3	0,55	30	4,1	0,41	30
**40**	4,43	0,47	40	3,38	0,28	40	3,9	0,44	30	2,87	0,3	30
**45**	3,3	0,37	40	2,5	0,25	40	2,8	0,43	30	2,17	0,26	30
**50**	2,3	0,24	40	2,05	0,21	40	2,17	0,38	30	1,63	0,23	30

Fig 4G

Dendritic branch points

	Scr. RNAi	RNAi#1	RNAi#1/JMY*	RNAi#1/JMY* ΔCA	RNAi#1/JMY*^{W981A}
	7	5	11	4	4
	8	3	15	4	3
	11	6	18	5	2
	9	4	16	4	5
	11	5	16	6	3
	7	9	14	4	3
	9	4	17	2	2
	11	6	20	6	5
	12	7	12	5	3
	8	4	20	6	3
	11	3	15	6	3
	9	5	13	3	3
	12	4	16	5	4
	15	6	12	5	2
	9	6	19	3	4
	6	6	8	2	2
	7	2	11	4	2
	7	2	12	4	2
	7	3	9	5	5
	6	4	9	3	2
	6	4	5	5	4
	5	2	10	4	3
	7	7	11	3	3
	7	3	10	5	3
	8	2	8	3	3
	4	2	12	3	4
	4	5	7	5	2
	3	4	17	4	1
	4	2	12	5	4
	7	2	12	4	2
	6	5	16	3	
	10	6	13	3	
	9	7	13	3	
	7	4	13	3	
	11	5	14	4	
	10	7	12	4	
	9	8	12	3	
	8	6	10	3	
	12	4	12	4	
	8	4	15	3	
	9	5	10	3	
	10	6	14	4	
	11	5	9	4	
	10	6	12	4	
	10	4	11	3	
	5	4	16		
	7	4	12		
	5	4	11		
	4	4	11		
	6	2	12		
	10	4	14		
	3	4	11		
	7	4	14		
	2	5	13		
	7	2	8		
	8	3	11		
	5	7	13		
	6	2	9		
	5	3	16		
	7	3	10		
	8	4	25		
	7	2	13		
	8	4	15		
	8	3	14		
	7	4	12		
	7	4	19		
	7	4	15		
	5	4	14		
	8	3	21		
	5	4	18		
	5	3	22		
	7	6	20		
	7	4	17		
	6	4	20		
	10	5	20		
**mean**	7,59	4,29	13,59	3,96	3,03
**SD**	2,44	1,57	3,83	1,04	1,03
**n**	75	75	75	45	30
**SEM**	0,28	0,18	0,44	0,16	0,19

Fig 4H

Dendritic terminal points

Scr. RNAi	RNAi#1	RNAi#1/JMY*	RNAi#1/JMY* ΔCA	RNAi#1/JMY*^{W981A}
21	18	21	15	21
21	17	33	18	14
28	19	37	16	20
28	16	25	20	21
30	18	39	21	20
18	22	25	16	11
28	18	35	14	20
25	24	33	14	18
25	21	25	18	17
26	21	34	25	16
31	22	31	17	11
23	24	24	16	16
29	15	29	17	23
35	19	25	17	15
29	18	32	14	17
25	22	23	20	16
19	17	26	15	17
22	14	21	16	15
25	15	22	22	21
22	15	25	13	17
24	19	18	21	22
23	12	24	17	16
27	18	26	13	12
20	16	23	21	14
25	11	24	16	15
19	13	28	16	18
22	18	21	20	16
19	24	35	11	11
21	13	31	15	17
23	17	26	19	12
22	20	32	21	
20	21	29	11	
25	21	30	14	
17	19	22	16	
29	18	24	15	
19	18	28	14	
24	18	26	11	
25	24	21	19	
27	17	23	20	
21	23	27	13	
26	20	23	21	
20	18	29	20	
27	24	19	21	
23	21	25	19	
23	13	24	15	
19	20	29		
26	21	25		
20	19	24		
14	23	18		
16	18	23		
20	17	33		
17	19	29		
23	15	29		
13	20	23		
20	10	15		
24	14	26		
17	16	21		
21	10	27		
21	24	31		
28	14	20		
21	14	43		
19	16	27		
22	21	29		
27	20	29		
19	15	27		
26	16	33		
25	17	37		
24	12	25		
26	15	33		
22	19	30		
21	16	35		
19	18	38		
23	19	37		
20	16	35		
21	18	37		
22,87	17,91	27,61	16,96	16,63
4,03	3,45	5,65	3,27	3,36
75	75	75	45	30
0,47	0,40	0,65	0,49	0,61

Fig 4I

Total dendritic length [µm]

Scr. RNAi	RNAi#1	RNAi#1/JMY*	RNAi#1/JMY* ΔCA	RNAi#1/JMY*^{W981A}
593,25	556,4	701,25	608,63	733,35
603,67	820,2	1023,49	681,02	483,8
1063,08	735,85	1172,87	605,36	768,67
876,7	610,97	995,87	813,45	640,04
1268,56	784,96	1569,47	716,78	872,18
655,95	901,25	944,89	569,57	403,64
1076,9	635,08	1429,75	481,17	726,46
852,57	824,31	898,67	510,76	734,14
969,55	841,46	871,78	782,63	656,05
694,31	847,65	935,7	904,88	501,71
993,2	1023,18	1175,41	828,26	371,52
1051,81	880,2	892,48	466,01	647,82
1124,47	639,43	854,22	765,31	823,32
1279,4	724,61	888,7	1001,72	555,64
893,66	1037,39	932,55	594,46	685,03
1005,52	890,46	791,88	772,58	541,71
770,96	704,33	1014,74	617,05	692,58
1148,31	565,55	823,97	573,11	491,21
826,87	645,96	602,79	745,45	939,25
835	578,22	737,06	452,81	523,24
895,26	585,34	740,68	935,82	642,12
774,91	598,74	765,95	558,37	508,81
936,4	704,68	1025,86	484,46	317,86
843,86	638,44	887,69	915,98	463,13
1006,32	351,64	917,44	492,99	684,04
850,11	456,23	941,11	527,28	682,35
855,77	831,41	901,01	694,57	623,67
702,41	720,5	1450,58	381,98	355,74
808,74	580,09	1181,75	594,28	584,85
836,12	587,19	800,64	641,5	349,99
743,13	979,64	1035,1	709,46	
806,5	942,24	791,54	329,13	
922,64	1088,39	970,82	422,63	
577,27	736,94	988,49	602	
1335,36	680,1	689,45	650,53	
671,56	800,6	1161,71	636,38	
763,88	622,15	923,15	591,66	
1032,4	1013	751,86	798,26	
914,78	612,04	817,68	675,21	
635,49	873,77	826,92	461,57	
1051,54	802,67	729,26	775,86	
1213,04	784,38	1005,6	807,3	
778,25	911,44	712,26	746,12	
740,21	836,64	990,86	741,17	
636,33	432,2	1020,79	976,13	
667,47	747,67	951,22		
945,83	1144,55	878,18		
721,45	766,42	751,44		
685,51	1218,86	678,85		
693,28	690,5	707,98		
858,96	778,37	1201,01		
605,05	707,34	968,99		
825,17	692,33	924,2		
532,83	921,26	630,14		
963,19	423,23	674,42		
1100,85	526,65	948		
708,75	622,12	691,41		
648,15	508,71	791,24		
857,89	940,52	925,58		
989,81	472,08	627,07		
708,65	725,1	1403,76		
875,85	621,14	730,9		
699,23	893,94	927,54		
897,25	934,46	894,22		
726,76	597,68	922,07		
1021,51	775,57	1135,91		
873,58	753,69	1401,01		
809,96	569,62	801,37		
1097,11	591,08	1002,54		
995,03	873,48	997,61		
708,88	697,39	1290,65		
767,34	785,19	1391,38		
935,46	665,4	1370,64		
760,84	773,02	1236,07		
763,78	580,73	1171,47		
858,15	738,88	950,83	658,70	600,13
176,89	172,73	218,42	160,73	158,04
75	75	75	45	30
20,43	19,94	25,22	23,96	28,85

Fig 5A

Dendritic branch points

	Scr. RNAi	RNAi#1	RNAi#1/JMY*	RNAi#1/JMY*$^{WH2\#1-3mut}$
	7	4	19	3
	8	4	10	2
	7	1	14	3
	6	3	14	4
	10	5	13	3
	8	3	15	3
	6	4	15	3
	10	4	13	2
	9	1	13	2
	7	3	16	3
	7	3	10	1
	8	3	20	1
	6	3	16	2
	8	3	25	4
	7	3	13	2
	5	3	12	4
	7	4	10	3
	6	3	14	1
	6	4	13	2
	7	2	15	3
	5	3	9	3
	6	3	12	2
	5	3	15	4
	5	3	14	3
	4	2	15	2
	6	1	16	2
	7	3	12	2
	6	4	14	2
	6	2	17	2
	5	2	13	2
**mean**	6,67	2,97	14,23	2,50
**SD**	1,45	0,96	3,20	0,86
**n**	30	30	30	30
**SEM**	0,26	0,18	0,58	0,16

Fig 5B

Dendritic terminal points

Scr. RNAi	RNAi#1	RNAi#1/JMY*	RNAi#1/JMY*$^{WH2\#1-3mut}$
29	18	36	17
23	13	21	15
20	12	29	12
25	14	27	19
28	19	27	14
22	17	32	11
25	16	31	12
28	13	27	10
28	14	25	12
22	18	32	13
28	15	24	16
24	15	35	12
19	13	30	15
26	17	41	24
21	13	25	14
13	14	26	13
25	14	24	16
16	14	30	9
18	20	26	12
20	8	27	13
14	11	20	14
22	23	30	12
20	13	28	16
16	11	27	12
13	11	25	12
22	10	28	7
16	14	23	13
18	14	27	15
16	19	28	11
17	12	25	14
21,13	14,50	27,87	13,50
4,80	3,26	4,38	3,14
30	30	30	30
0,88	0,59	0,80	0,57

Fig 5C

Total dendritic length [µm]

Scr. RNAi	RNAi#1	RNAi#1/JMY*	RNAi#1/JMY*$^{WH2\#1-3mut}$
1009,13	649,77	1043,54	736,4
891,87	608,52	759,91	532,3
755,19	693,08	889,09	812,63
996,69	581,91	1126,44	695,76
879,01	920,79	1096,42	606,26
1097,69	760,83	1007,26	498,16
959	773,29	1080,06	492,53
880,59	436,85	868,23	423,88
1004,95	584,94	866,33	560,35
980,49	773,85	1061,03	422,99
988,81	669,46	908,26	493,86
911,11	705,17	1229,39	372,45
763,13	498,2	755,82	601,14
818,19	784,27	1258,44	988,4
897,31	645,94	954,56	634,22
424,42	746,03	716,7	543,09
837,76	526,79	599,17	505,1
407,62	572,05	1061,87	259,24
486,65	901,04	679,51	406,87
693,08	329,01	814,53	518,14
417,23	466,83	539,92	431,02
737,36	675,67	833,59	415,41
614,02	440,1	807,28	608,08
676,27	414,02	769,09	542,35
411,16	373,02	828,59	489,36
824,98	299,77	957,83	396,87
571,74	492,1	775,12	478,12
572,35	528,78	702,24	597,12
518,53	757,92	808,6	404,98
816,26	391,04	762,19	507,76
761,42	600,03	885,37	532,49
207,63	165,24	177,44	143,25
30	30	30	30
37,91	30,17	32,40	26,15

Fig 5H

Dendritic branch points

	Scr. RNAi	RNAi#1	RNAi#1/JMY*	RNAi#1/JMY* ΔCaM
	7	5	11	4
	8	3	15	5
	11	6	18	3
	9	4	16	4
	11	5	16	3
	7	9	14	3
	9	4	17	7
	11	6	20	5
	12	7	12	4
	8	4	20	5
	11	3	15	4
	9	5	13	5
	12	4	16	3
	15	6	12	3
	9	6	19	2
	6	6	8	5
	7	2	11	4
	7	2	12	7
	7	3	9	6
	6	4	9	3
	6	4	5	3
	5	2	10	3
	7	7	11	3
	7	3	10	5
	8	2	8	5
	4	2	12	4
	4	5	7	4
	3	4	17	2
	4	2	12	4
	7	2	12	2
**mean**	7,90	4,23	12,90	4,00
**SD**	2,77	1,83	3,92	1,31
**n**	30	30	30	30
**SEM**	0,51	0,33	0,71	0,24

Fig 5I

Dendritic terminal points

Scr. RNAi	RNAi#1	RNAi#1/JMY*	RNAi#1/JMY* ΔCaM
21	18	21	18
21	17	33	21
28	19	37	18
28	16	25	20
30	18	39	13
18	22	25	20
28	18	35	19
25	24	33	19
25	21	25	20
26	21	34	19
31	22	31	18
23	24	24	17
29	15	29	13
35	19	25	19
29	18	32	14
25	22	23	20
19	17	26	31
22	14	21	29
25	15	22	19
22	15	25	14
24	19	18	14
23	12	24	17
27	18	26	17
20	16	23	19
25	11	24	22
19	13	28	20
22	18	21	21
19	24	35	15
21	13	31	19
23	17	26	20
24,43	17,87	27,37	18,83
4,09	3,55	5,40	3,91
30	30	30	30
0,75	0,65	0,99	0,71

Fig 5J

Total dendritic length [µm]

Scr. RNAi	RNAi#1	RNAi#1/JMY*	RNAi#1/JMY* ΔCaM
593,25	556,4	701,25	719,61
603,67	820,2	1023,49	857,83
1063,08	735,85	1172,87	777,97
876,7	610,97	995,87	794,68
1268,56	784,96	1569,47	612,63
655,95	901,25	944,89	724,08
1076,9	635,08	1429,75	775,43
852,57	824,31	898,67	735,02
969,55	841,46	871,78	839,18
694,31	847,65	935,7	690,71
993,2	1023,18	1175,41	740,13
1051,81	880,2	892,48	848,16
1124,47	639,43	854,22	516,34
1279,4	724,61	888,7	745,02
893,66	1037,39	932,55	779,26
1005,52	890,46	791,88	609,28
770,96	704,33	1014,74	1244,97
1148,31	565,55	823,97	997,81
826,87	645,96	602,79	635,89
835	578,22	737,06	424,13
895,26	585,34	740,68	551,22
774,91	598,74	765,95	613,64
936,4	704,68	1025,86	493,86
843,86	638,44	887,69	568,28
1006,32	351,64	917,44	791,03
850,11	456,23	941,11	700,56
855,77	831,41	901,01	844,4
702,41	720,5	1450,58	511,25
808,74	580,09	1181,75	639,61
836,12	587,19	800,64	741,78
903,12	710,06	962,34	717,46
175,90	158,70	222,30	162,43
30	30	30	30
32,12	28,97	40,59	29,66

Fig 6B

t (min)	actin	GST	GST-Cobl WH2#2	GST-JMY WH2#1	GST-JMY WH2#2	GST-JMY WH2#3
0:00:00	0	0	0	0	0	0
0:00:05	5,139	5,499	-5,651	-3,093	-3,786	-0,82
0:00:10	11,158	13,46	-4,736	-6,296	-3,514	-0,962
0:00:15	18,201	18,518	-5,951	-5,683	-3,631	3,541
0:00:20	20,86	22,259	-8,093	-2,927	-1,853	1,348
0:00:25	27,728	31,104	-12,23	-4,931	8,115	3,518
0:00:30	35,237	36,354	-17,153	-5,78	10,447	2,219
0:00:35	43,004	44,793	-16,075	-4,229	10,397	5,16
0:00:40	47,914	48,853	-14,892	-3,166	8,27	8,154
0:00:45	55,14	54,844	-18,621	-1,644	6,544	7,015
0:00:50	60,482	64,657	-16,635	-1,81	9,714	9,1
0:00:55	66,824	74,106	-16,926	-1,171	10,251	10,686
0:01:00	72,072	82,272	-17,945	-4,836	13,711	10,608
0:01:05	82,831	87,96	-19,286	2,732	12,519	12,162
0:01:10	90,79	97,145	-22,642	-2,234	16,109	10,713
0:01:15	94,084	106,104	-21,197	2,89	12,802	9,867
0:01:20	98,247	109,802	-25,218	-0,661	15,736	12,402
0:01:25	103,665	120,49	-21,416	1,468	16,161	14,047
0:01:30	108,954	123,366	-28,803	4,46	18,352	14,705
0:01:35	117,845	135,953	-19,366	4,396	17,348	16,701
0:01:40	124,565	141,25	-26,875	5,052	19,166	21,59
0:01:45	132,683	145,655	-27,734	3,095	18,357	18,496
0:01:50	139,228	156,494	-25,51	5,179	21,541	23,405
0:01:55	144,406	160,932	-21,592	6,416	19,618	22,838
0:02:00	148,415	171,186	-24,656	8,58	21,455	24,352
0:02:05	154,945	178,224	-24	9,753	21,852	27,927
0:02:10	161,01	184,026	-27,249	10,486	21,633	29,058
0:02:15	168,667	197,227	-23,235	11,833	23,498	31,561
0:02:20	175,039	200,429	-19,907	9,969	24,449	34,111
0:02:25	183,55	211,031	-22,706	12,397	25,871	33,712
0:02:30	189,603	215,312	-21,999	14,729	24,661	37,555
0:02:35	192,471	225,385	-19,363	16,358	27,311	38,907
0:02:40	196,203	228,98	-21,216	16,313	26,37	39,117
0:02:45	205,691	238,935	-18,257	15,232	27,755	40,411
0:02:50	214,144	245,211	-16,473	16,624	26,987	43,474
0:02:55	220,121	255,274	-17,133	21,111	29,275	44,593
0:03:00	229,879	261,63	-19,938	16,227	28,983	44,946
0:03:05	232,24	273,135	-20,501	20,256	28,158	48,85
0:03:10	238,93	274,064	-22,389	19,927	32,879	50,818
0:03:15	249,098	282,916	-18,329	24,403	33,176	55,122
0:03:20	251,845	293,293	-21,864	24,465	30,926	53,917
0:03:25	259,008	302,999	-15,671	21,941	30,132	59,3
0:03:30	262,605	303,888	-14,475	27,776	30,048	61,162
0:03:35	267,96	313,053	-18,539	27,005	36,93	62,537
0:03:40	279,861	321,449	-16,513	27,224	33,225	63,219
0:03:45	286,66	330,899	-14,141	29,549	33,841	64,902
0:03:50	291,617	335,281	-11,336	27,702	34,983	66,451
0:03:55	293,14	346,907	-11,957	32,385	34,81	69,303
0:04:00	305,582	359,149	-13,278	35,169	35,548	73,077
0:04:05	309,549	359,764	-15,171	33,801	37,54	74,217
0:04:10	316,626	370,045	-11,097	33,606	36,93	77,069
0:04:15	318,892	376,975	-10,724	36,266	33,964	77,857
0:04:20	328,367	382,427	-6,671	37,42	36,886	80,125
0:04:25	332,732	391,232	-5,7	39,961	39,446	81,099
0:04:30	336,196	394,164	-5,556	39,42	36,821	84,55
0:04:35	344,016	401,933	-7,017	38,9	36,18	86,627
0:04:40	350,858	413,506	-3,768	40,094	37,297	92,537
0:04:45	355,505	416,767	-3,173	44,835	41,448	94,806
0:04:50	360,511	428,147	-2,928	41,949	39,376	94,368
0:04:55	369,585	427,728	-1,317	49,527	40,429	98,068
0:05:00	373,498	442,247	0,098	46,471	36,389	99,237
0:05:05	375,986	446,242	0,489	49,205	41,601	103,284
0:05:10	386,618	452,131	2,307	46,917	44,729	104,453
0:05:15	396,418	461,101	3,033	51,046	38,613	104,199
0:05:20	398,271	468,477	3,746	53,342	41,901	107,327
0:05:25	402,392	475,276	7,615	54,796	42,506	107,868
0:05:30	412,469	480,98	6,324	54,446	41,846	112,351
0:05:35	417,868	486,758	9,269	56,722	41,378	112,846
0:05:40	423,927	496,903	11,905	59,211	41,614	116,621
0:05:45	430,222	502,203	10,031	60,411	41,733	117,955
0:05:50	438,216	513,998	12,783	60,846	43,522	123,533
0:05:55	445,354	513,646	11,563	64,687	43,444	123,824
0:06:00	444,33	527,752	17,989	63,512	46,002	126,503
0:06:05	455,268	530,95	18,528	58,74	41,664	130,835
0:06:10	460,688	536,148	20,035	63,277	41,315	132,73
0:06:15	468,286	543,076	18,797	65,227	41,573	134,117
0:06:20	461,48	550,054	19,002	64,324	46,286	137,214
0:06:25	477,437	556,033	21,693	69,206	44,355	137,698
0:06:30	481,112	565,986	18,731	66,904	42,278	142,751
0:06:35	489,448	568,391	21,281	70,837	45,972	144,684
0:06:40	491,912	576,837	22,853	72,113	43,084	144,324
0:06:45	502,408	586,295	27,602	71,059	46,906	150,172
0:06:50	507,52	592,99	29,716	74,461	45,61	150,236
0:06:55	507,627	593,819	27,499	78,239	42,338	150,453
0:07:00	515,19	600,131	29,802	78,736	48,188	154,518
0:07:05	521,471	609,177	26,987	76,515	45,325	162,519
0:07:10	529,487	613,416	33,885	83,814	46,301	159,026
0:07:15	531,964	622,244	36,997	78,04	46,576	162,641
0:07:20	539,312	629,291	32,116	81,218	46,804	167,057
0:07:25	547,045	635,62	37,831	82,093	45,962	164,391
0:07:30	546,841	642,287	37,381	85,381	46,832	168,595
0:07:35	556,334	646,517	39,771	84,977	46,737	174,455
0:07:40	559,684	652,974	40,911	85,34	47,437	176,285
0:07:45	568,471	661,216	39,05	85,499	44,71	174,374
0:07:50	571,913	668,582	42,95	89,905	47,324	178,57
0:07:55	577,044	672,97	43,344	87,881	47,196	177,83
0:08:00	582,951	679,627	46,653	93,441	49,413	183,342
0:08:05	592,529	684,247	47,245	92,449	48,716	187,468
0:08:10	593,414	689,666	48,206	94,619	48,091	188,638
0:08:15	602,156	691,856	46,919	96,635	46,901	194,326
0:08:20	601,423	701,1	49,384	98,823	49,501	196,193
0:08:25	615,034	706,875	55,362	100,799	49,359	195,667
0:08:30	611,797	710,389	54,711	103,954	50,695	203,088
0:08:35	617,233	716,175	56,09	100,159	50,584	201,277
0:08:40	623,81	722,482	58,419	104,796	49,239	205,621
0:08:45	633,273	730,747	60,189	107,425	51,248	208,788
0:08:50	635,72	738,951	58,206	106,763	50,419	207,142
0:08:55	641,891	734,779	59,52	104,805	52,053	213,925
0:09:00	651,492	748,479	60,056	110,482	52,971	216,242
0:09:05	647,308	752,444	63,236	110,783	55,813	217,462
0:09:10	659,559	760,874	68,068	115,359	53,146	220,256
0:09:15	666,956	766,788	69,052	113,417	50,418	222,086
0:09:20	669,148	775,884	69,863	116,24	51,942	225,494
0:09:25	678,053	780,529	69,662	115,757	51,578	224,466
0:09:30	678,175	783,082	70,73	116,942	54,208	232,608
0:09:35	683,74	788,701	75,964	118,237	55,904	227,564
0:09:40	700,81	789,065	73,905	117,919	52,897	232,647
0:09:45	695,429	794,508	74,949	120,82	54,348	235,202
0:09:50	698,638	799,405	79,228	118,337	55,349	236,482
0:09:55	708,713	805,474	76,887	126,088	53,697	241,089
0:10:00	712,021	816,733	78,053	126,454	56,867	243,853

Fig 6D

	GFP-JMY CT	GFP-JMY WH2#1	GFP-JMY WH2#2	GFP-JMY WH2#3
	100	1,60	0	30,39
	100	6,10	0	42,12
	100	44,20	0	0
	100	21,58	0	5,40
	100	98,48	0,44	0,04
	100	0	10,93	0
	100	69,60	0,80	2,29
	100	488,25		
	100	0,17		
**mean**	100,00	81,11	1,74	11,46
**SD**	0,00	156,60	4,06	17,38
**n**	9	9	7	7
**SEM**	0,00	52,20	1,54	6,57

Fig 6F

Dendritic branch points

	Scr. RNAi	RNAi#1	RNAi#1/JMY*	RNAi#1/JMY*$^{WH2\#1mut}$
	7	4	19	6
	8	4	10	4
	7	1	14	4
	6	3	14	3
	10	5	13	2
	8	3	15	3
	6	4	15	5
	10	4	13	4
	9	1	13	4
	7	3	16	2
	7	3	10	3
	8	3	20	4
	6	3	16	2
	8	3	25	2
	7	3	13	3
	5	3	12	3
	7	4	10	3
	6	3	14	2
	6	4	13	4
	7	2	15	2
	5	3	9	2
	6	3	12	4
	5	3	15	2
	5	3	14	2
	4	2	15	3
	6	1	16	3
	7	3	12	3
	6	4	14	2
	6	2	17	2
	5	2	13	4
**mean**	6,67	2,97	14,23	3,07
**SD**	1,45	0,96	3,20	1,05
**n**	30	30	30	30
**SEM**	0,26	0,18	0,58	0,19

Fig 6G

Dendritic terminal points

Scr. RNAi	RNAi#1	RNAi#1/JMY*	RNAi#1/JMY*$^{WH2\#1mut}$
29	18	36	23
23	13	21	17
20	12	29	11
25	14	27	16
28	19	27	16
22	17	32	20
25	16	31	20
28	13	27	20
28	14	25	17
22	18	32	16
28	15	24	17
24	15	35	15
19	13	30	17
26	17	41	13
21	13	25	15
13	14	26	15
25	14	24	16
16	14	30	8
18	20	26	16
20	8	27	12
14	11	20	10
22	23	30	12
20	13	28	10
16	11	27	10
13	11	25	13
22	10	28	18
16	14	23	14
18	14	27	13
16	19	28	11
17	12	25	19
21,13	14,50	27,87	15,00
4,80	3,26	4,38	3,58
30	30	30	30
0,88	0,59	0,80	0,65

Fig 6H

Total dendritic length [µm]

Scr. RNAi	RNAi#1	RNAi#1/JMY*	RNAi#1/JMY*$^{WH2\#1mut}$
1009,13	649,77	1043,54	930,56
891,87	608,52	759,91	571,42
755,19	693,08	889,09	638,36
996,69	581,91	1126,44	639,92
879,01	920,79	1096,42	778,28
1097,69	760,83	1007,26	718,86
959	773,29	1080,06	805,32
880,59	436,85	868,23	825,38
1004,95	584,94	866,33	816,97
980,49	773,85	1061,03	550,65
988,81	669,46	908,26	572,75
911,11	705,17	1229,39	737,27
763,13	498,2	755,82	835,76
818,19	784,27	1258,44	563,47
897,31	645,94	954,56	806,58
424,42	746,03	716,7	551,11
837,76	526,79	599,17	698,73
407,62	572,05	1061,87	241,01
486,65	901,04	679,51	593,82
693,08	329,01	814,53	428,29
417,23	466,83	539,92	384,82
737,36	675,67	833,59	632,25
614,02	440,1	807,28	366,84
676,27	414,02	769,09	291,19
411,16	373,02	828,59	397,68
824,98	299,77	957,83	774,29
571,74	492,1	775,12	426,62
572,35	528,78	702,24	429,06
518,53	757,92	808,6	276,2
816,26	391,04	762,19	783,37
761,42	600,03	885,37	602,23
207,63	165,24	177,44	190,93
30	30	30	30
37,91	30,17	32,40	34,86

Fig 6J

Coprecipitated CaM	$+Ca^{2+}$
	415,7
	194,62
	540,09
**mean**	383,47
**SD**	174,98
**n**	3
**SEM**	101,02

Coprecipitated Actin	$+Ca^{2+}$
	-88,18
	-48,08
	-93,39
	-76,55
	24,79
	3
	14,31

Fig 6L

	EGTA	Ca^{2+}	EGTA	Ca^{2+}
	**GFP-JMY CT**	**GFP-JMY CT**	**GFP-JMY WH2#1**	**GFP-JMY WH2#1**
	0	-94,65	0	-99,54
	0	-97,77	0	-83,97
	0	-91,87	0	-99,92
	0	-95,85		
	0	-66,58	0	-55,89
**mean**	0,00	-89,34	0,00	-84,83
**SD**	0,00	12,90	0,00	20,67
**n**	5	5	4	4
**SEM**	0,00	5,77	0,00	10,34

Fig 6M

	1 mM EGTA	2 µM Ca^{2+}	2 µM Ca^{2+}/ 1mM EGTA (subsequently)
	0	-88,52	-69,78
	0	-88,49	-68,42
	0	-66,19	6,93
	0	-88,2	-20,49
	0	-66,22	
	0	-91,83	
**mean**	0,00	-81,58	-37,94
**SD**	0,00	11,98	37,69
**n**	6	6	4
**SEM**	0,00	4,89	18,84

Fig 7B

Dendritic branch points

	GFP			JMY*/GFP		
	Mean	SEM	n	Mean	SEM	n
**DMSO**	5,93	0,18	40	10,35	0,36	40
**CGS9342B**	3	0,21	30	2,67	0,19	30

Fig 7C

Dendritic terminal points

	GFP			JMY*/GFP		
	Mean	SEM	n	Mean	SEM	n
**DMSO**	17,73	0,56	40	23,58	0,55	40
**CGS9342B**	15,7	0,8	30	14,37	0,61	30

Fig 7D

Total dendritic length [µm]

	GFP			JMY*/GFP		
	Mean	SEM	n	Mean	SEM	n
**DMSO**	623,46	25,14	40	716,6	22,13	40
**CGS9342B**	543,26	34,47	30	505,19	30,65	30

Fig 7E

Sholl analyses

Sholl	GFP+DMSO			GFP+CGS9343B			JMY*/GFP+DMSO			JMY*/GFP+CGS9343B		
intersections	Mean	SEM	n	Mean	SEM	n	Mean	SEM	n	Mean	SEM	n
**10**	12,8	0,52	40	13,23	0,73	30	15,75	0,6	40	12,27	0,53	30
**15**	15,38	0,56	40	14,23	0,79	30	20,25	0,77	40	13,13	0,55	30
**20**	15,23	0,61	40	13,2	0,84	30	18,88	0,68	40	12,53	0,62	30
**25**	12,45	0,69	40	10	0,8	30	12,83	0,62	40	9,33	0,7	30
**30**	9,05	0,66	40	7,17	0,71	30	7,85	0,52	40	5,7	0,6	30
**35**	5,93	0,53	40	4,5	0,61	30	4,83	0,42	40	3,53	0,41	30
**40**	4,43	0,47	40	3,23	0,53	30	3,38	0,28	40	2,43	0,33	30
**45**	3,3	0,37	40	2,27	0,38	30	2,5	0,25	40	1,8	0,23	30
**50**	2,3	0,24	40	1,47	0,26	30	2,05	0,21	40	1,47	0,21	30

Fig S1C

	0 µM	12,5 nM	25 nM	50 nM	100 nM
	0,0	51,7	60,5	9,2	32,5
	0,0	100	100	37,4	70,8
	0,0	102,3	96,0	97,8	43,5
	0,0			69,2	83,7
	0,0			96,0	100
	0,0			93,0	99,2
**mean**	0,00	84,68	85,51	67,12	71,62
**SD**	0,00	28,59	21,72	36,56	28,39
**n**	6	3	3	6	6
**SEM**	0,00	16,50	12,54	14,93	11,59

Fig S1G

	**JMY*/GFP**	GFP
	80	20
	70	18
	64	38
	78	24
**mean**	73,00	25,00
**SD**	7,39	9,02
**n**	4	4
**SEM**	3,70	4,51

Fig S2B

	Scr. RNAi	RNAi#1
	1,47	0,66
	0,95	0,64
	0,92	0,98
	0,98	0,95
	0,99	0,59
	0,97	0,75
	1,03	0,75
	0,58	0,68
	1	0,48
	0,99	0,56
	1,02	0,55
	0,95	0,62
	1,06	0,82
	1,05	0,76
**mean**	1,00	0,70
**SD**	0,18	0,15
**n**	14	14
**SEM**	0,05	0,04

Fig S2D

Dendritic branch points

	Scr. RNAi	RNAi#2	RNAi#2/JMY*
	6	9	17
	10	10	12
	9	11	14
	7	10	15
	11	8	11
	10	8	12
	9	10	13
	8	9	11
	12	8	14
	8	3	9
	9	8	11
	10	4	12
	11	2	15
	10	9	10
	10	5	8
	1	5	13
	4	0	14
	5	4	12
	7	3	16
	8	6	12
	8	1	28
	2	3	21
	9	6	14
	19	4	14
	19	5	21
	12	3	16
	5	1	32
	10	18	16
	21	14	24
	8	10	43
	17	4	21
	20	10	25
	11	6	14
	10	10	11
	11	6	9
	19	9	
	22	9	
	12	4	
	24	5	
	23	6	
	15	5	
	17	3	
	19	13	
	15	8	
		10	
		5	
		4	
		7	
		6	
		6	
**mean**	11,66	6,66	16,00
**SD**	5,68	3,54	7,22
**n**	44	50	35
**SEM**	0,86	0,50	1,22

Fig S2E

Dendritic terminal points

Scr. RNAi	RNAi#2	RNAi#2/JMY*
22	21	26
20	26	26
25	22	23
17	24	28
29	20	22
19	31	27
24	27	28
25	30	21
27	26	27
21	17	23
26	25	20
20	18	28
27	20	26
23	20	25
23	13	17
6	19	23
15	6	25
20	16	21
14	18	29
19	18	25
21	9	45
18	11	38
20	17	33
43	16	25
29	19	34
22	14	43
16	10	51
21	26	37
37	32	42
14	26	64
26	19	33
33	19	39
20	16	27
21	19	29
16	18	24
39	21	
30	26	
20	15	
27	14	
31	17	
25	14	
25	14	
27	26	
26	21	
	23	
	16	
	16	
	16	
	25	
	17	
23,39	19,38	30,11
6,85	5,65	9,75
44	50	35
1,03	0,80	1,65

Fig S2F

Total dendritic length [µm]

Scr. RNAi	RNAi#2	RNAi#2/JMY*
743,13	881,87	932,1
806,5	812,4	1102,72
922,64	1064,35	817,14
577,27	888,17	897,06
1335,36	939,9	626,18
671,56	1095,29	844,4
763,88	860,31	993,11
1032,4	1281,85	565,12
914,78	883,43	921,59
635,49	724,22	679,1
1051,54	827,33	709,27
1213,04	565,56	1073,94
778,25	761,78	945,74
740,21	679,22	870,56
636,33	543,2	617,07
204,07	819,13	775,79
491,07	220,13	1054,11
898,43	535,97	881,28
693,62	699,59	881,67
817,05	657,77	1002,83
683,14	377,09	1609,66
689,33	370,01	1422
685,7	528,67	1229,28
1224,67	577,44	975,65
1137,76	784,44	1300,16
665,11	493,38	1355,71
547,78	464,33	2045,23
846,25	1144,24	1365,96
1268,9	1324,43	1633,27
476,79	992,78	2064,32
1335,51	651,34	1493,4
1099,3	585,15	1660,4
697,74	449,45	906,32
773,81	1034,46	767,79
467,98	559,45	1097,11
1275,19	716,55	
1009,5	857,47	
738,95	627,89	
780,53	676,05	
1070,75	486,77	
905,82	676,05	
948,01	505,76	
1180,66	982,49	
630,37	628,62	
	618,2	
	637,51	
	660,88	
	473,25	
	1260,27	
	592,49	
842,41	729,57	1089,06
260,47	245,49	380,13
44	50	35
39,27	34,72	64,25

Fig S2G

Depth analyses

	Scr. RNAi			RNAi#2			RNAi#2/JMY*		
**Depth**	**Mean**	**SEM**	**n**	**Mean**	**SEM**	**n**	**Mean**	**SEM**	**n**
**1**	10,45	0,59	51	11,2	0,84	35	13,69	0,78	36
**2**	10,96	0,74	51	11	0,82	35	13,89	0,78	36
**3**	6,33	0,54	51	3,71	0,4	35	8,03	0,81	36
**4**	3,16	0,52	51	1	0,28	35	4,89	0,59	36
**5**	1,29	0,34	51	0,17	0,1	35	2,11	0,36	36
**6**	0,94	0,37	51	0,06	0,06	35	1,28	0,3	36
**7**	0,53	0,27	51	0	0	35	0,83	0,26	36
**8**	0,27	0,17	51	0	0	35	0,33	0,19	36

Fig S2H

Sholl analyses

Sholl	Scr. RNAi			RNAi#2			RNAi#2/JMY*		
**intersections**	**Mean**	**SEM**	**n**	**Mean**	**SEM**	**n**	**Mean**	**SEM**	**n**
**10**	13	0,57	44	12,56	0,55	50	15,83	0,86	35
**15**	16,45	0,88	44	15,02	0,66	50	21,03	0,95	35
**20**	18,07	0,89	44	16,08	0,82	50	21,94	1,07	35
**25**	16,25	0,85	44	13,7	0,8	50	17,8	1,11	35
**30**	13,09	0,86	44	10,52	0,71	50	12,8	0,88	35
**35**	9,86	0,72	44	8,16	0,65	50	9,71	0,84	35
**40**	6,82	0,62	44	5,96	0,51	50	7,03	0,77	35
**45**	5,27	0,52	44	4,5	0,42	50	5,86	0,67	35
**50**	4,11	0,46	44	3,2	0,41	50	5	0,6	35
**55**	3,34	0,39	44	2,8	0,45	50	4,74	0,57	35

Fig S3B

Sholl analyses

**Sholl intersections**	**Scr. RNAi**			**RNAi#1**			**RNAi#1/JMY***			**RNAi#1/JMY* ΔCT**			**RNAi#1/JMY* ΔNT**		
	**Mean**	**SEM**	**n**	**Mean**	**SEM**	**n**	**Mean**	**SEM**	**n**	**Mean**	**SEM**	**n**	**Mean**	**SEM**	**n**
**10**	15,21	0,4	75	12,93	0,39	75	16,04	0,41	75	13,51	0,41	45	13,82	0,57	45
**15**	17,73	0,51	75	14,11	0,41	75	21,65	0,63	75	15,2	0,47	45	15,13	0,55	45
**20**	18,49	0,48	75	14,43	0,41	75	22,24	0,59	75	15,56	0,57	45	15,78	0,6	45
**25**	15,59	0,48	75	12,44	0,43	75	18,85	0,58	75	14	0,58	45	13,47	0,66	45
**30**	11,61	0,47	75	9,91	0,42	75	13,45	0,56	75	11,13	0,63	45	9,49	0,6	45
**35**	7,84	0,41	75	7,15	0,36	75	8,93	0,45	75	8,8	0,62	45	6,24	0,44	45
**40**	5,39	0,33	75	5,29	0,31	75	6,16	0,35	75	6,36	0,47	45	4,07	0,28	45
**45**	4	0,26	75	4,19	0,3	75	4,53	0,28	75	4,73	0,43	45	2,78	0,19	45
**50**	3,15	0,23	75	3,13	0,25	75	3,32	0,24	75	3,78	0,35	45	2,07	0,17	45
**55**	2,47	0,2	75	2,61	0,22	75	2,73	0,22	75	3,16	0,28	45	1,53	0,16	45
**60**	2,15	0,2	75	2,32	0,19	75	2,36	0,2	75	2,49	0,24	45	1,16	0,11	45

Fig S4B

Sholl analyses

Sholl intersections	Scr. RNAi			RNAi#1			RNAi#1/JMY*			RNAi#1/JMY* ΔCA			RNAi#1/JMY*^{W981A}		
	Mean	SEM	n	Mean	SEM	n	Mean	SEM	n	Mean	SEM	n	Mean	SEM	n
**10**	16,8	0,48	30	13,9	0,53	30	15,9	0,61	30	13,24	0,45	45	13,77	0,54	30
**15**	19,77	0,55	30	15,23	0,6	30	21,13	0,82	30	14,73	0,51	45	15,4	0,58	30
**20**	21,1	0,71	30	15,9	0,7	30	22,63	0,79	30	15,38	0,51	45	14,67	0,61	30
**25**	17,87	0,97	30	13,4	0,56	30	18,2	0,76	30	12,24	0,53	45	11,07	0,66	30
**30**	13,53	1,04	30	9,9	0,52	30	12,6	0,74	30	8,27	0,47	45	7,17	0,59	30
**35**	9,37	0,86	30	6,73	0,46	30	8,57	0,54	30	5,53	0,36	45	4,67	0,52	30
**40**	6,23	0,69	30	4,93	0,42	30	6,1	0,48	30	3,91	0,28	45	3,1	0,38	30
**45**	4,33	0,48	30	3,3	0,34	30	4,6	0,45	30	2,96	0,24	45	2,3	0,31	30
**50**	3,5	0,39	30	2,73	0,35	30	3,7	0,35	30	2,18	0,23	45	1,73	0,24	30

Fig S5B

Sholl analyses

Sholl intersections	Scr. RNAi			RNAi#1			RNAi#1/JMY*			RNAi#1/JMY*$^{WH2\#1\text{-}3mut}$		
	**Mean**	**SEM**	**n**	**Mean**	**SEM**	**n**	**Mean**	**SEM**	**n**	**Mean**	**SEM**	**n**
**10**	15,37	0,69	30	11,9	0,51	30	16,6	0,59	30	11,13	0,5	30
**15**	18,43	0,89	30	12,83	0,53	30	22,87	0,9	30	12,17	0,52	30
**20**	18,6	0,92	30	13,03	0,56	30	22,37	0,87	30	11,9	0,64	30
**25**	14,8	0,92	30	10,87	0,56	30	17,4	0,81	30	9,07	0,59	30
**30**	10,53	0,68	30	8,07	0,58	30	12,07	0,78	30	6,47	0,51	30
**35**	6,6	0,6	30	6,17	0,53	30	8,03	0,71	30	5,03	0,44	30
**40**	4,7	0,46	30	4,73	0,44	30	5,27	0,46	30	3,5	0,36	30
**45**	3,77	0,41	30	3,63	0,36	30	3,97	0,38	30	3,1	0,34	30
**50**	2,93	0,35	30	2,67	0,3	30	3	0,37	30	2,47	0,32	30
**55**	2,33	0,3	30	2,03	0,27	30	2,4	0,32	30	1,83	0,27	30
**60**	2,03	0,27	30	1,77	0,25	30	2,3	0,35	30	1,6	0,28	30

Fig S5E

Sholl analyses

Sholl intersections	Scr. RNAi			RNAi#1			RNAi#1/JMY*			RNAi#1/JMY* ΔCaM		
	Mean	SEM	n	Mean	SEM	n	Mean	SEM	n	Mean	SEM	n
**10**	16,8	0,48	30	13,9	0,53	30	15,9	0,61	30	14,9	0,63	30
**15**	19,77	0,55	30	15,23	0,6	30	21,13	0,82	30	16,5	0,69	30
**20**	21,1	0,71	30	15,9	0,7	30	22,63	0,79	30	16,33	0,68	30
**25**	17,87	0,97	30	13,4	0,56	30	18,2	0,76	30	13,57	0,7	30
**30**	13,53	1,04	30	9,9	0,52	30	12,6	0,74	30	9,2	0,53	30
**35**	9,37	0,86	30	6,73	0,46	30	8,57	0,54	30	6,17	0,49	30
**40**	6,23	0,69	30	4,93	0,42	30	6,1	0,48	30	4,27	0,42	30
**45**	4,33	0,48	30	3,3	0,34	30	4,6	0,45	30	3,23	0,37	30
**50**	3,5	0,39	30	2,73	0,35	30	3,7	0,35	30	2,67	0,32	30

Fig S6C

Sholl analyses

Sholl intersections	Scr. RNAi			RNAi#1			RNAi#1/JMY*			RNAi#1/JMY*$^{WH2\#1mut}$		
	**Mean**	**SEM**	**n**	**Mean**	**SEM**	**n**	**Mean**	**SEM**	**n**	**Mean**	**SEM**	**n**
**10**	15,37	0,69	30	11,9	0,51	30	16,6	0,59	30	12,2	0,56	30
**15**	18,43	0,89	30	12,83	0,53	30	22,87	0,9	30	13,03	0,61	30
**20**	18,6	0,92	30	13,03	0,56	30	22,37	0,87	30	12,97	0,66	30
**25**	14,8	0,92	30	10,87	0,56	30	17,4	0,81	30	10,27	0,55	30
**30**	10,53	0,68	30	8,07	0,58	30	12,07	0,78	30	7,83	0,56	30
**35**	6,6	0,6	30	6,17	0,53	30	8,03	0,71	30	5,77	0,53	30
**40**	4,7	0,46	30	4,73	0,44	30	5,27	0,46	30	4,23	0,39	30
**45**	3,77	0,41	30	3,63	0,36	30	3,97	0,38	30	3,37	0,35	30
**50**	2,93	0,35	30	2,67	0,3	30	3	0,37	30	2,67	0,33	30
**55**	2,33	0,3	30	2,03	0,27	30	2,4	0,32	30	2,37	0,33	30
**60**	2,03	0,27	30	1,77	0,25	30	2,3	0,35	30	1,93	0,3	30

Fig S6F

	EGTA	Ca^{2+}
	0	11,96
	0	-17,05
	0	16,9
	0	-2,42
**mean**	0,00	2,35
**SD**	0,00	15,31
**n**	4	4
**SEM**	0,00	7,65
