# Supplementary figures and images for: JMY powers dendritogenesis and is regulated by CaM revealing a general, critical principle in neuromorphogenesis
Source: Commun Biol. 2025 May 22;8:784. doi: 10.1038/s42003-025-08208-3 (PMC12098658; doi:10.1038/s42003-025-08208-3)

**Figure 1**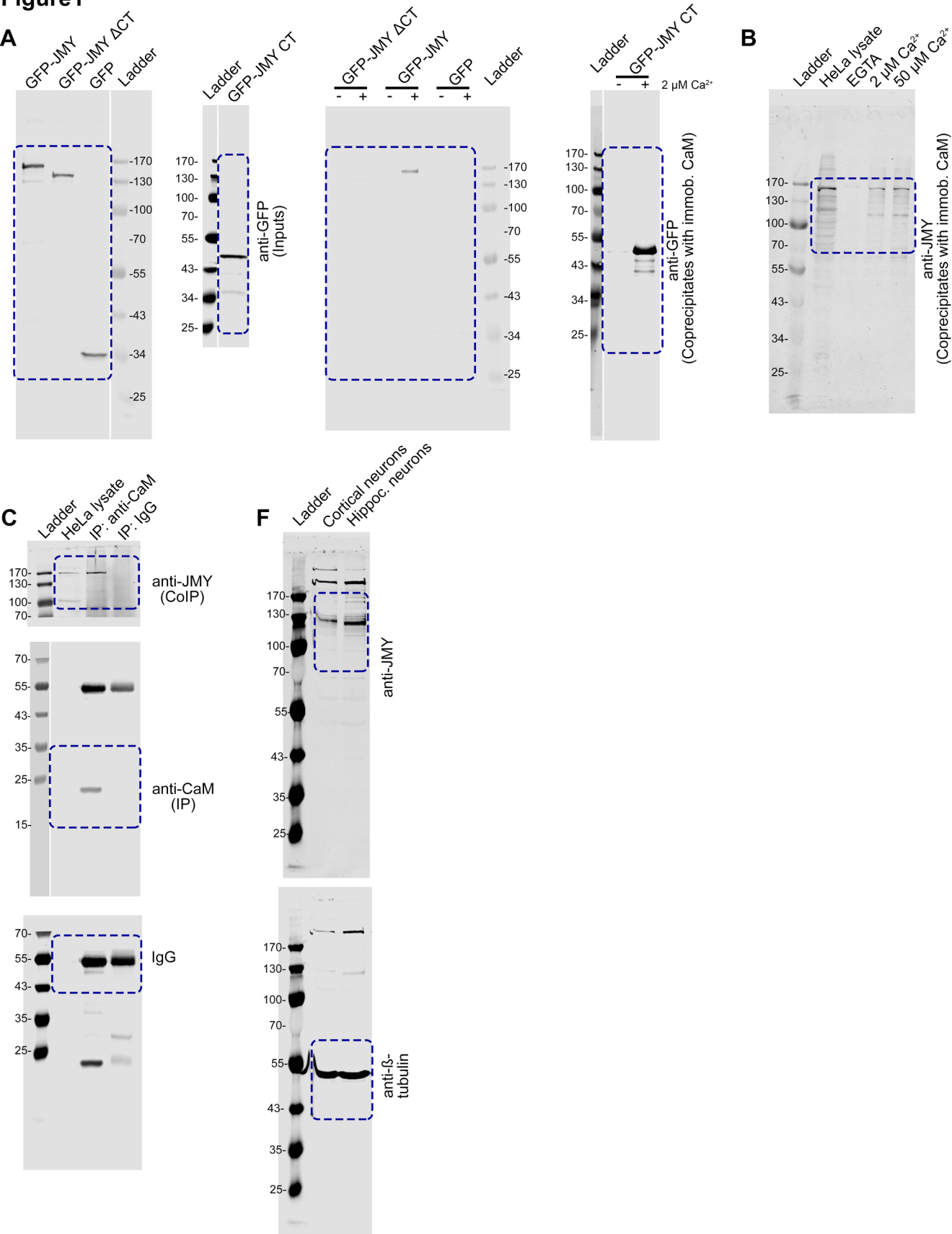

**Figure S1**

**B**

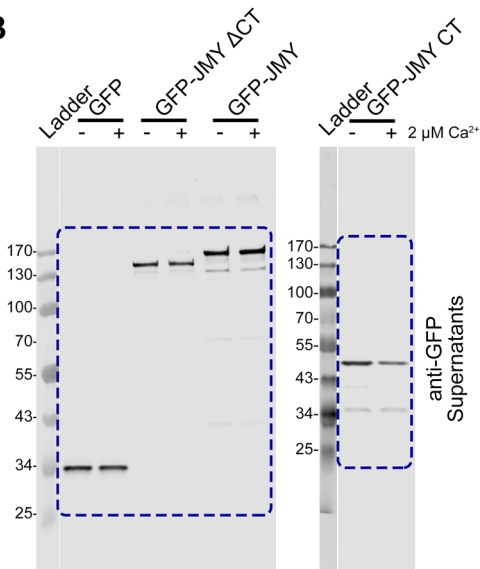

**Figure 5**

**E**

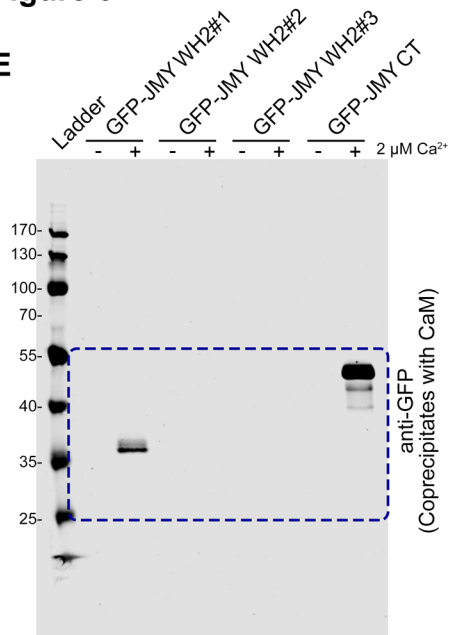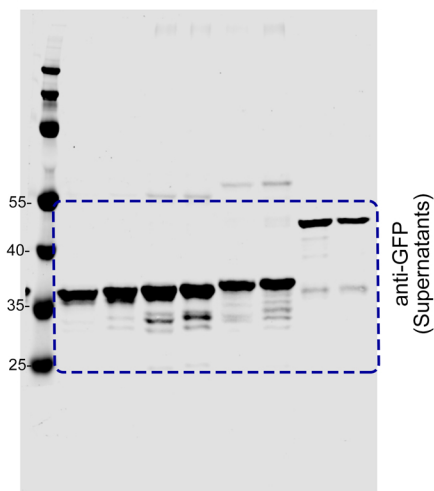

**F**

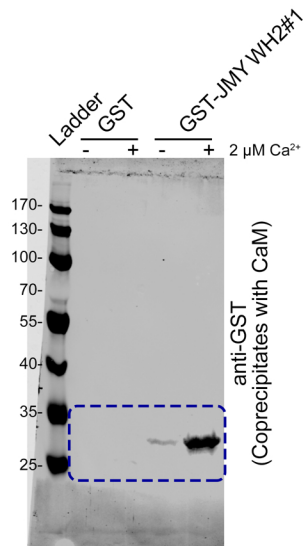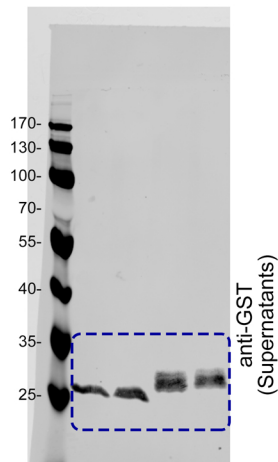

**Figure-S5**

**C**

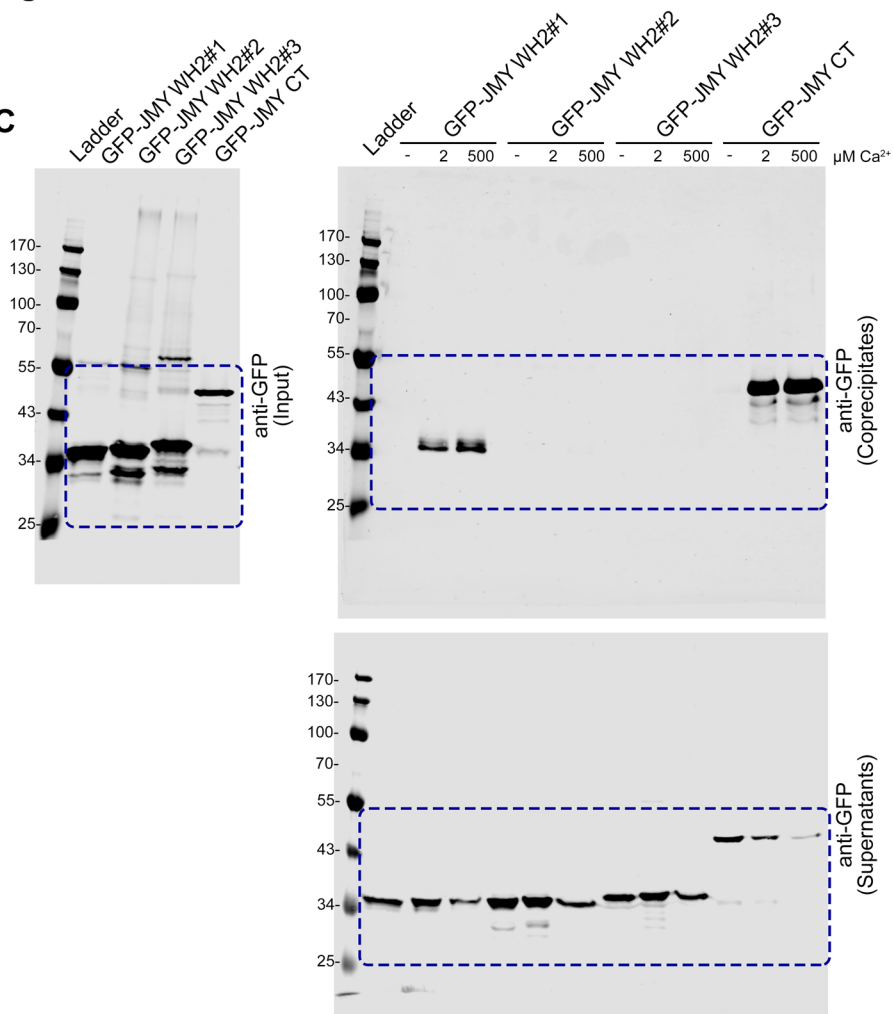

**D**

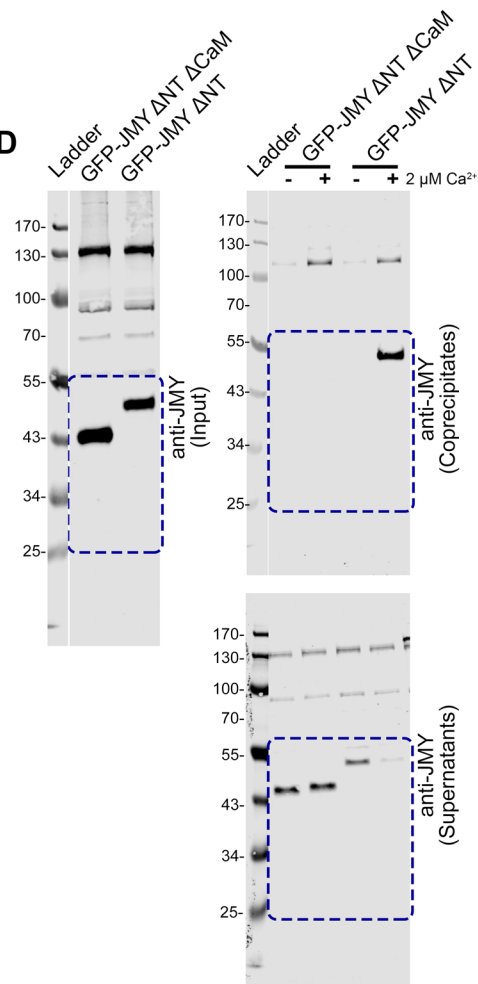

**Figure 6**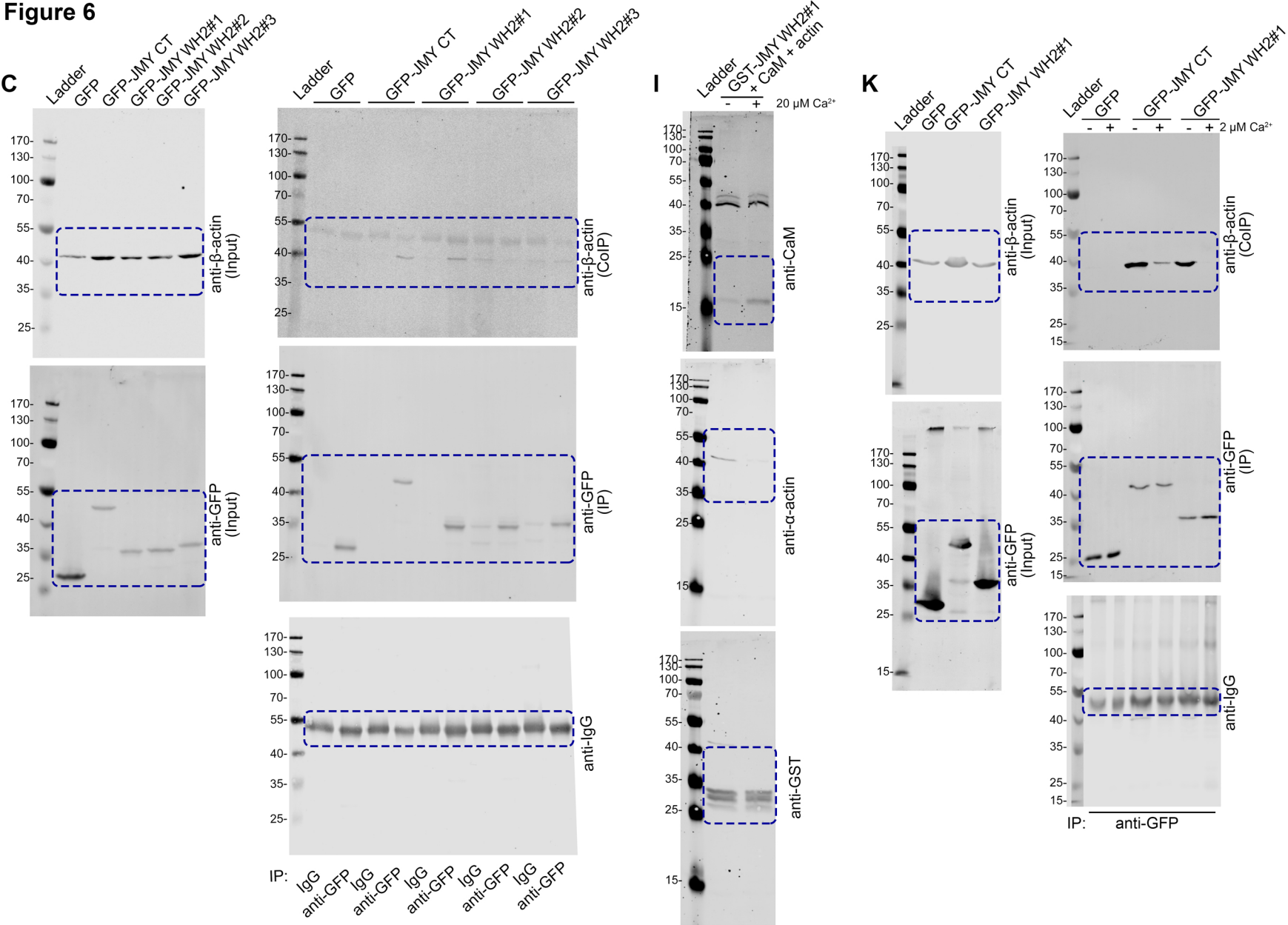

**Figure-S6**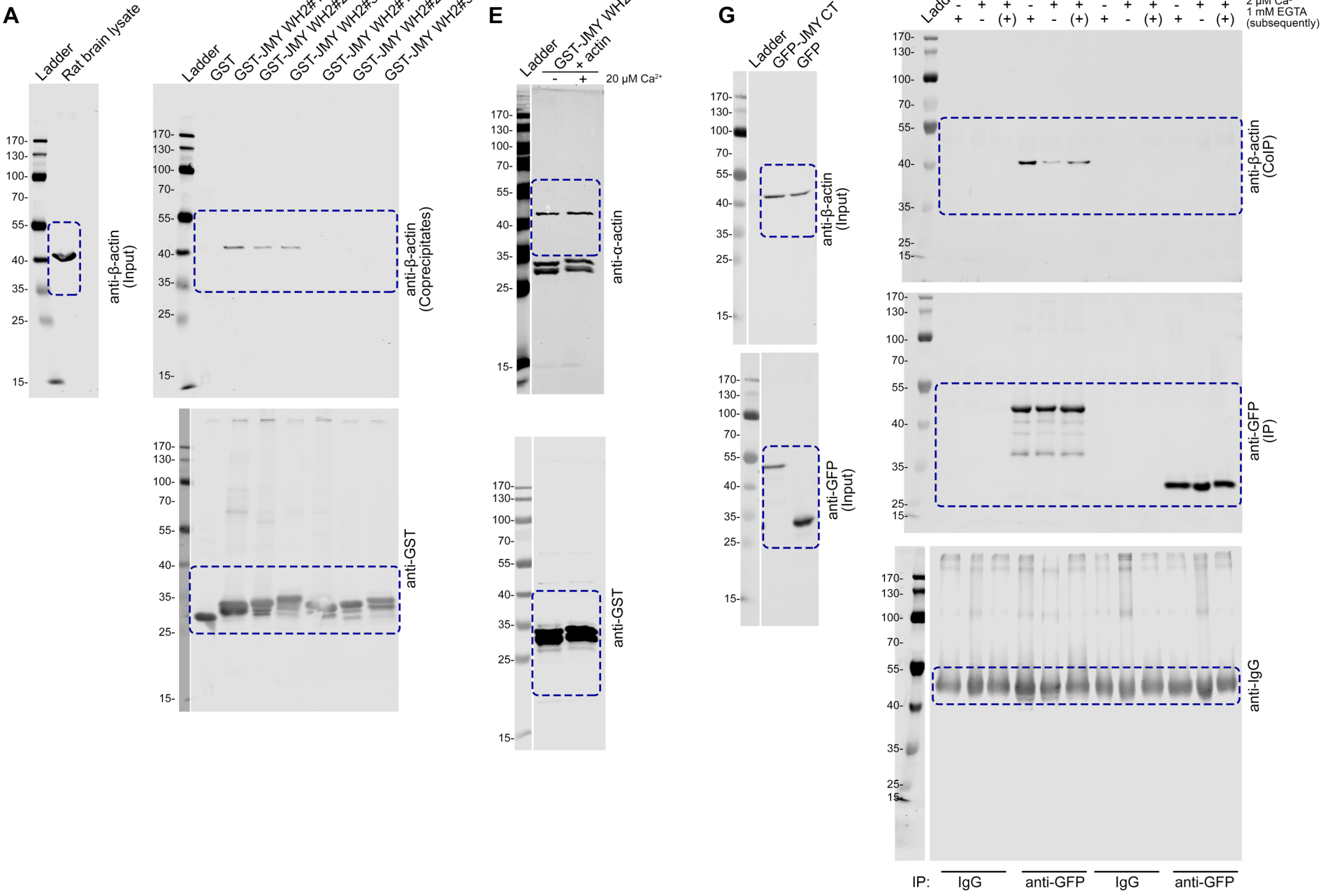

Supplement: Supplementary file 5 — Supplementary Data 2 [file 42003_2025_8208_MOESM5_ESM.pdf]
