# Supplementary material for: JMY powers dendritogenesis and is regulated by CaM revealing a general, critical principle in neuromorphogenesis
Source: Commun Biol. 2025 May 22;8:784. doi: 10.1038/s42003-025-08208-3 (PMC12098658; doi:10.1038/s42003-025-08208-3)
Supplement: Supplementary file 6 — Reporting Summary [file 42003_2025_8208_MOESM6_ESM.pdf]

nature portfolio

Corresponding author(s): Britta Qualmann
Michael M. Kessels
Maryam Izadi-Seitz

Last updated by author(s): 27.04.2025

Reporting Summary

Nature Portfolio wishes to improve the reproducibility of the work that we publish. This form provides structure for consistency and transparency in reporting. For further information on Nature Portfolio policies, see our Editorial Policies and the Editorial Policy Checklist.

Statistics

For all statistical analyses, confirm that the following items are present in the figure legend, table legend, main text, or Methods section.

n/a	Confirmed	
☐	☒	The exact sample size (*n*) for each experimental group/condition, given as a discrete number and unit of measurement
☐	☒	A statement on whether measurements were taken from distinct samples or whether the same sample was measured repeatedly
☐	☒	The statistical test(s) used AND whether they are one- or two-sided<br>*Only common tests should be described solely by name; describe more complex techniques in the Methods section.*
☐	☒	A description of all covariates tested
☐	☒	A description of any assumptions or corrections, such as tests of normality and adjustment for multiple comparisons
☐	☒	A full description of the statistical parameters including central tendency (e.g. means) or other basic estimates (e.g. regression coefficient) AND variation (e.g. standard deviation) or associated estimates of uncertainty (e.g. confidence intervals)
☐	☐	For null hypothesis testing, the test statistic (e.g. *F*, *t*, *r*) with confidence intervals, effect sizes, degrees of freedom and *P* value noted<br>*Give P values as exact values whenever suitable.*
☐	☐	For Bayesian analysis, information on the choice of priors and Markov chain Monte Carlo settings
☐	☐	For hierarchical and complex designs, identification of the appropriate level for tests and full reporting of outcomes
☐	☐	Estimates of effect sizes (e.g. Cohen's *d*, Pearson's *r*), indicating how they were calculated

Our web collection on statistics for biologists contains articles on many of the points above.

Software and code

Policy information about availability of computer code

Data collection	only commercially available software or Freeware was used and all software used is listed in the manuscript
Data analysis	only commercially available software or Freeware was used and all software used is listed in the manuscript

For manuscripts utilizing custom algorithms or software that are central to the research but not yet described in published literature, software must be made available to editors and reviewers. We strongly encourage code deposition in a community repository (e.g. GitHub). See the Nature Portfolio guidelines for submitting code & software for further information.

Data

Policy information about availability of data

All manuscripts must include a data availability statement. This statement should provide the following information, where applicable:

- Accession codes, unique identifiers, or web links for publicly available datasets
- A description of any restrictions on data availability
- For clinical datasets or third party data, please ensure that the statement adheres to our policy

The authors declare that all data supporting the findings of this study are available within the paper and its supplementary information files.

Research involving human participants, their data, or biological material

Policy information about studies with human participants or human data. See also policy information about sex, gender (identity/presentation), and sexual orientation and race, ethnicity and racism.

Reporting on sex and gender	Does not apply
Reporting on race, ethnicity, or other socially relevant groupings	*Please specify the socially constructed or socially relevant categorization variable(s) used in your manuscript and explain why they were used. Please note that such variables should not be used as proxies for other socially constructed/relevant variables (for example, race or ethnicity should not be used as a proxy for socioeconomic status).*<br>*Provide clear definitions of the relevant terms used, how they were provided (by the participants/respondents, the researchers, or third parties), and the method(s) used to classify people into the different categories (e.g. self-report, census or administrative data, social media data, etc.)*<br>*Please provide details about how you controlled for confounding variables in your analyses.*
Population characteristics	*Describe the covariate-relevant population characteristics of the human research participants (e.g. age, genotypic information, past and current diagnosis and treatment categories). If you filled out the behavioural & social sciences study design questions and have nothing to add here, write "See above."*
Recruitment	*Describe how participants were recruited. Outline any potential self-selection bias or other biases that may be present and how these are likely to impact results.*
Ethics oversight	*Identify the organization(s) that approved the study protocol.*

Note that full information on the approval of the study protocol must also be provided in the manuscript.

Field-specific reporting

Please select the one below that is the best fit for your research. If you are not sure, read the appropriate sections before making your selection.

☒ Life sciences ☐ Behavioural & social sciences ☐ Ecological, evolutionary & environmental sciences

For a reference copy of the document with all sections, see nature.com/documents/nr-reporting-summary-flat.pdf

Life sciences study design

All studies must disclose on these points even when the disclosure is negative.

Sample size	No statistical methods were used to predetermine sample size.<br>Sampling of neurons was done in a systematic manner. Therefore, the n numbers merely reflect the outcome of the systematic searches and differ (30-75 neurons). N-numbers, numbers of independent assays and statistical significance analyses are reported in the figure legends. Neuronal morphology evaluations are always based on cells of at least two independent neuronal preparations (2-5 independent preparations).
Data exclusions	No "outlier" suggestions were computed. No data points were excluded, but all technically sound quantitative evaluation data points were taken into account and averaged to fully represent biological and methodological variabilities.
Replication	All quantitative neuronal data is derived of at least two independent neuronal preparations.<br>Also all work with cell lines is based on at least two independent cell passages, seedings and transfections.<br>All data shown in form of examples have been reproduced.
Randomization	There was no allocation of samples into experimental groups except for the fact that certain rows in a cell culture plate were incubated with a given reagent and other rows with something else. As within one assay anyway all conditions were analyzed, i.e. no data/samples were excluded, and the cellular preparation is the same in all cell culture wells and several wells/coverslips were imaged for transfected cells, randomization of samples is not a concern in our study.
Blinding	Analyses were conducted in a non-blinded manner, as neuronal sampling was systematic and all neuronal evaluations were done in an automated manner (e.g. 3D reconstructions IMARIS software and exclusively software-based extraction of dendritic parameters from the IMARIS 3D reconstruction ("filament").

Behavioural & social sciences study design

All studies must disclose on these points even when the disclosure is negative.

Study description	*Briefly describe the study type including whether data are quantitative, qualitative, or mixed-methods (e.g. qualitative cross-sectional, quantitative experimental, mixed-methods case study).*
Research sample	*State the research sample (e.g. Harvard university undergraduates, villagers in rural India) and provide relevant demographic*

Research sample	*information (e.g. age, sex) and indicate whether the sample is representative. Provide a rationale for the study sample chosen. For studies involving existing datasets, please describe the dataset and source.*
Sampling strategy	*Describe the sampling procedure (e.g. random, snowball, stratified, convenience). Describe the statistical methods that were used to predetermine sample size OR if no sample-size calculation was performed, describe how sample sizes were chosen and provide a rationale for why these sample sizes are sufficient. For qualitative data, please indicate whether data saturation was considered, and what criteria were used to decide that no further sampling was needed.*
Data collection	*Provide details about the data collection procedure, including the instruments or devices used to record the data (e.g. pen and paper, computer, eye tracker, video or audio equipment) whether anyone was present besides the participant(s) and the researcher, and whether the researcher was blind to experimental condition and/or the study hypothesis during data collection.*
Timing	*Indicate the start and stop dates of data collection. If there is a gap between collection periods, state the dates for each sample cohort.*
Data exclusions	*If no data were excluded from the analyses, state so OR if data were excluded, provide the exact number of exclusions and the rationale behind them, indicating whether exclusion criteria were pre-established.*
Non-participation	*State how many participants dropped out/declined participation and the reason(s) given OR provide response rate OR state that no participants dropped out/declined participation.*
Randomization	*If participants were not allocated into experimental groups, state so OR describe how participants were allocated to groups, and if allocation was not random, describe how covariates were controlled.*

Ecological, evolutionary & environmental sciences study design

All studies must disclose on these points even when the disclosure is negative.

Study description	*Briefly describe the study. For quantitative data include treatment factors and interactions, design structure (e.g. factorial, nested, hierarchical), nature and number of experimental units and replicates.*
Research sample	*Describe the research sample (e.g. a group of tagged Passer domesticus, all Stenocereus thurberi within Organ Pipe Cactus National Monument), and provide a rationale for the sample choice. When relevant, describe the organism taxa, source, sex, age range and any manipulations. State what population the sample is meant to represent when applicable. For studies involving existing datasets, describe the data and its source.*
Sampling strategy	*Note the sampling procedure. Describe the statistical methods that were used to predetermine sample size OR if no sample-size calculation was performed, describe how sample sizes were chosen and provide a rationale for why these sample sizes are sufficient.*
Data collection	*Describe the data collection procedure, including who recorded the data and how.*
Timing and spatial scale	*Indicate the start and stop dates of data collection, noting the frequency and periodicity of sampling and providing a rationale for these choices. If there is a gap between collection periods, state the dates for each sample cohort. Specify the spatial scale from which the data are taken*
Data exclusions	*If no data were excluded from the analyses, state so OR if data were excluded, describe the exclusions and the rationale behind them, indicating whether exclusion criteria were pre-established.*
Reproducibility	*Describe the measures taken to verify the reproducibility of experimental findings. For each experiment, note whether any attempts to repeat the experiment failed OR state that all attempts to repeat the experiment were successful.*
Randomization	*Describe how samples/organisms/participants were allocated into groups. If allocation was not random, describe how covariates were controlled. If this is not relevant to your study, explain why.*
Blinding	*Describe the extent of blinding used during data acquisition and analysis. If blinding was not possible, describe why OR explain why blinding was not relevant to your study.*

Did the study involve field work? ☐ Yes ☐ No

Field work, collection and transport

Field conditions	*Describe the study conditions for field work, providing relevant parameters (e.g. temperature, rainfall).*
Location	*State the location of the sampling or experiment, providing relevant parameters (e.g. latitude and longitude, elevation, water depth).*
Access & import/export	*Describe the efforts you have made to access habitats and to collect and import/export your samples in a responsible manner and in compliance with local, national and international laws, noting any permits that were obtained (give the name of the issuing authority, the date of issue, and any identifying information).*

Disturbance	*Describe any disturbance caused by the study and how it was minimized.*

Reporting for specific materials, systems and methods

We require information from authors about some types of materials, experimental systems and methods used in many studies. Here, indicate whether each material, system or method listed is relevant to your study. If you are not sure if a list item applies to your research, read the appropriate section before selecting a response.

Materials & experimental systems

n/a	Involved in the study	
☐	☒	Antibodies
☐	☒	Eukaryotic cell lines
☒	☐	Palaeontology and archaeology
☐	☒	Animals and other organisms
☒	☐	Clinical data
☒	☐	Dual use research of concern
☒	☐	Plants

Methods

n/a	Involved in the study	
☒	☐	ChIP-seq
☒	☐	Flow cytometry
☒	☐	MRI-based neuroimaging

Antibodies

Antibodies used	Rabbit anti-JMY antibodies directed against the C terminal part of JMY applied for detecting the expression and localization of untagged JMY* ΔNT were from Proteintech Group, Inc. (25098-1-AP). Rabbit anti-JMY antibodies direct against the N terminal part of JMY were from Antibodies-online GmbH (ABIN2838360). Polyclonal rabbit anti-GFP (ab290) and polyclonal chicken anti-GFP (ab13970) antibodies as well as monoclonal mouse anti-Arp3 antibodies (ab49671) were from Abcam. Monoclonal mouse anti-GFP antibodies (JL-8) were from Clontech (632381). Monoclonal anti-β-actin (A5441), anti-α-actin (A2066), anti-MAP2 (HM-2; M4403) and anti-β-tubulin antibodies (T4026) were from Sigma. Monoclonal mouse anti-CaM (G3) antibodies were from Santa Cruz Biotechnology. Affinity-purified rabit and guinea pig anti-GST antibodies were described previously.<br>Alexa Fluor 568-conjugated phalloidin and MitoTracker Deep Red 633 were from Molecular Probes.<br>Secondary antibodies used included Alexa Fluor 568-labeled donkey anti-mouse antibodies (R37114 and A10037), Alexa Fluor 680-labeled goat anti-mouse (A-21236 and A32723) and anti-rabbit antibodies (AB_2535758), Alexa Fluor-labeled 488 donkey anti–rabbit (A-21206), Alexa Fluor 680-labeled donkey anti-rabbit (AB_2922888), goat anti-rabbit antibodies coupled to IRDye800 (AB_2556775) and DyLight800-conjugated goat anti–mouse antibodies (AB_2556774) were from Thermo Fisher Scientific. Donkey anti-guinea pig antibodies coupled to IRDye680 (925-32411) and donkey Anti-chicken IRDye 800CW (925-32218) were from LI-COR Bioscience. Goat anti-chicken Alexa Fluor 680 were from Abcam.
Validation	Affinity-purified rabit and guinea pig anti-GST antibodies were described previously (Qualmann et al., 1999, Braun et al., 2005 and subsequent publications).<br>The commerical antibodies were mostly not validated further, as they gave rise to proper bands of overexpressed proteins (anti-tag antibodies anti-GFP, anti-mcherry, anti-Flag) or bands and subcellular patterns of the expectedxpected sizes and nature (anti-MAP2, anti-actin, anti-GAPDH) and/or have been used in publications before.<br>Secondary antibodies were validated by specific signals of different primary antibodies detected.

Eukaryotic cell lines

Policy information about cell lines and Sex and Gender in Research

Cell line source(s)	HEK293 cells, human embryonic kidney, were ordered from Cell Lines Services GmbH, Eppelheim, DE.<br>HeLa cells were ordered from Cell Lines Services GmbH, Eppelheim, DE.<br>L929 cells were from the DSMZ.<br>COS7 cells were ordered from Cell Lines Services GmbH, Eppelheim, DE.
Authentication	HEK293 cells were authenticated by a STR profiling (conducted by Microsynth, Balgach, Switzerland; last authentication order and certificate 10/2017). STR profiling-authenticated set of vials are still in stock and fresh ones are taken into culture upon high passage numbers.<br>The other cell lines were not authenticated further but were as obtained from the commercial providers.
Mycoplasma contamination	All cell lines are regularly tested for mycoplasma contaminations and were negative.
Commonly misidentified lines<br>(See ICLAC register)	No commonly misidentified cell line was used

Animals and other research organisms

Policy information about studies involving animals; ARRIVE guidelines recommended for reporting animal research, and Sex and Gender in Research

Laboratory animals	Rats (Crl:WI; Charles River) were used for primary cell culture preparations from brain tissue.<br>Animal housing and breeding was done by the animal facility of University Hospital Jena in strict compliance with the EU directives 86/609/EWG and 2007/526/EG guidelines for animal experiments. All procedures were approved by the local government (Thüringer Landesamt, Bad Langensalza, Germany, and Landesverwaltungsamt; breeding allowance UKJ-17-021; allowance for primary hippocampal culture preparations from rat embryos (UKJ-24-005)).
Wild animals	none
Reporting on sex	mixed (ratio undetermined), as embryos were used and the hippocampi of all embryos of one mother animal were pooled for the generation of each primary cell culture.<br>Brains from WT mice for biochemical examinations (coprecipitation analyses, coimmunoprecipitations) were from bmice of mixed sex as well.
Field-collected samples	none
Ethics oversight	No specific ethical oversight required. Tissue material use from sacrificed animals only.

Note that full information on the approval of the study protocol must also be provided in the manuscript.

Plants

Seed stocks	*Report on the source of all seed stocks or other plant material used. If applicable, state the seed stock centre and catalogue number. If plant specimens were collected from the field, describe the collection location, date and sampling procedures.*
Novel plant genotypes	*Describe the methods by which all novel plant genotypes were produced. This includes those generated by transgenic approaches, gene editing, chemical/radiation-based mutagenesis and hybridization. For transgenic lines, describe the transformation method, the number of independent lines analyzed and the generation upon which experiments were performed. For gene-edited lines, describe the editor used, the endogenous sequence targeted for editing, the targeting guide RNA sequence (if applicable) and how the editor was applied.*
Authentication	*Describe any authentication procedures for each seed stock used or novel genotype generated. Describe any experiments used to assess the effect of a mutation and, where applicable, how potential secondary effects (e.g. second site T-DNA insertions, mosiacism, off-target gene editing) were examined.*
